# Progress in Stereoselective Haloamination of Olefins

**DOI:** 10.3390/molecules30153217

**Published:** 2025-07-31

**Authors:** Guo Zhong, Jiayu Zhou, Bin Cui, Hui Sun

**Affiliations:** Manganese Catalysis and Asymmetric Synthesis Laboratory, College of Chemistry and Pharmaceutical Engineering, Hebei University of Science and Technology, Shijiazhuang 050018, China; 19856940637@163.com (G.Z.); zhoujiayu0724@163.com (J.Z.)

**Keywords:** intermolecular, intramolecular, stereoselective haloamination, catalysis, olefins

## Abstract

The regio- and stereoselective adjacent bifunctionalization of olefins with amine and halogen groups can be effectively accomplished through catalytic haloamination methods. Stereoselective haloamination has emerged as a pivotal methodology for the introduction of halogen functional groups into chiral amines, demonstrating substantial applications in medicinal chemistry and organic synthesis. Since 1999, significant advancements have been achieved in this field, driven by innovations in catalytic systems and methodologies. The stereoselective haloamination of both functionalized and nonfunctionalized alkenes employing chiral catalysts has emerged as a prominent area of research. This review provides a comprehensive overview of the research progress in stereoselective haloamination reactions from 1999 to 2023. It examines the innovations in catalyst design that have facilitated more efficient and selective transformations. The review also analyzes the optimization of reaction conditions, which has been crucial in improving the overall performance and applicability of these reactions. Furthermore, it explores the diverse range of haloamination reactions that have been developed, emphasizing their potential for the synthesis of complex and valuable chemical structures. Additionally, this review offers insightful perspectives on future research directions in stereoselective haloamination reactions.

## 1. Introduction

Olefinic compounds often undergo halogenation and amination reactions with halogen-containing compounds (such as X = F, Cl, Br, and I) and nitrogen sources, representing a direct method for the synthesis of vicinal haloamines [1,2]. Vicinal haloamines are widely acknowledged as versatile building blocks in the fields of organic and medicinal chemistry [3], serving as key intermediates for subsequent transformations [4]. Under specific conditions, these intermediates have the potential to be transformed into nitrogen-containing heterocyclic compounds [5,6]. Haloamination reactions can be classified into intramolecular and intermolecular haloamination processes. It is noteworthy that the pioneering intramolecular haloamination reactions of amino alkenes were conducted over a century ago [7,8]. The key mechanistic steps in haloamination reactions are generally considered to involve the formation of either nitrogen-centered cationic intermediates [9,10,11,12,13] or halonium ions. Three predominant reaction mechanisms have been well-established:

(i) The electrophilic haloamination mechanism, wherein halonium ions (X^+^) function as electrophiles to attack alkenes, forming halonium ion intermediates that subsequently undergo nucleophilic addition by amine compounds;
(ii) The radical-mediated mechanism, where halogen radicals (X^•^) generated through radical initiators or photoredox catalysis initiate radical addition to alkenes;
(iii) The intramolecular reaction mechanism, which proceeds either through a concerted transition state enabling simultaneous formation of C-X and C-N bonds, or via cyclization of substrates containing pre-installed halogen and amine functional groups to construct heterocyclic frameworks.

The substitution reactions of amino groups can proceed via either mono- or polysubstitution pathways, with the specific reaction outcome being determined by both the reaction conditions and the structural features of the participating reactants. Haloamination continues to pose significant challenges in modern organic and medicinal chemistry [14,15,16,17], with its products, o-haloamines, serving as crucial precursors for a diverse array of compounds, including azopyridines [18,19,20] and dehydroamino acids [21].

The achievement of stereoselective haloamination reactions critically depends on catalytic systems, which can be broadly classified into two categories: organocatalysts and transition-metal-based catalysts. The latter includes metals such as iron [22,23], silver [24], copper [25,26], and palladium [27,28], among others. The nitrogen sources for stereoselective haloamination reactions are typically derived from amines, sulfonamides, carbamates, and amides [29,30,31,32,33], whereas the primary halogen sources consist of p-chlorobenzenesulfonimide [34] and N-iodosuccinimide (NIS). With continuous advancements in research, the substrate scope of stereoselective haloamination reactions has expanded considerably. Functionalized olefins, including α,β-unsaturated esters [35], amides, nitriles, and ketones, have exhibited exceptional stereoselectivity under optimized reaction conditions. Stereoselective haloamination has advanced significantly through the development of various methodologies. Although metal catalysts exhibit relatively high activity in facilitating stereoselective haloamination, they are often expensive and contribute to significant environmental pollution. For instance, nickel and copper catalysis demonstrate high activity but necessitate efficient ligands and incur substantial costs [36]. In contrast, non-metal catalysts are environmentally friendly and align with the principles of green chemistry. Overall, each method presents distinct advantages and disadvantages. It is essential to consider the nature of the substrate, the reaction conditions, and the desired products when selecting the most appropriate methodology.

At present, the research in the field of stereoselective haloamination has not been systematically sorted out, and therefore there is an urgent need for a comprehensive and in-depth summary of its research results. In this paper, the reaction mechanism of stereoselective haloamination and the unique advantages of each reaction are reviewed in detail, aiming to provide a valuable reference to help the development of this reaction in the field of organic synthesis and to support the construction of more diversified reaction types. To elucidate the stereochemical control in haloamination reactions, this study systematically analyzes both non-stereoselective transformations and their stereoselective counterparts, categorized as general (nonselective), intramolecular stereoselective, and intermolecular stereoselective haloamination processes. This review systematically summarizes recent advances in stereoselective haloamination reactions, elucidates their underlying mechanistic pathways, and provides perspectives on future research directions, aiming to facilitate further developments in this field and enhance its applications in organic synthesis.

## 2. General Haloamination

**Scheme 1:**
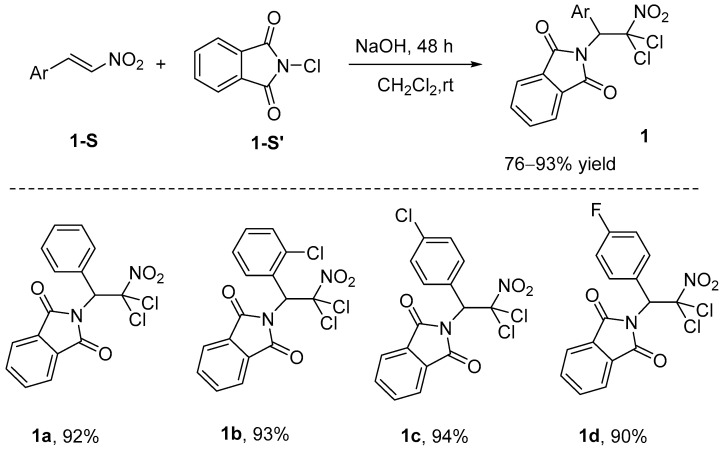
N-Chlorophthalimide as nitrogen source for haloamination reaction.

**Scheme 2:**
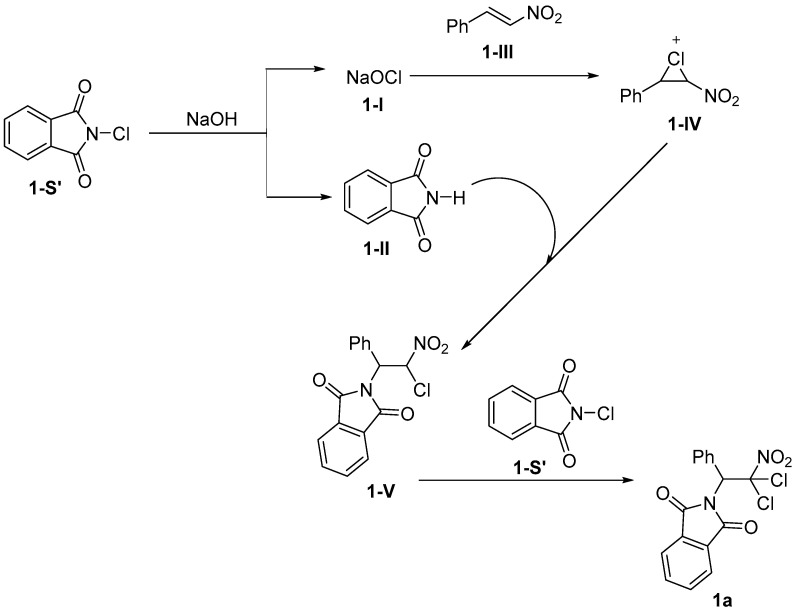
Reaction mechanism of N-chlorophthalimide as nitrogen source for haloamination reaction.

In recent years, significant advancements have been made in the field of haloamination and related reactions. These reactions not only enhance the synthetic efficiency of compounds but broaden the scope of their applications. For example, Qian et al. [37] demonstrated that N-chlorophthalimide functions as an efficient dual-source reagent for nitrogen and chlorine, exhibiting exceptional compatibility with a wide range of β-nitrophenyl substrates. The reaction exhibits a high yield and demonstrates excellent regioselectivity. This process takes 48 h to complete, and shortening the reaction time decreases the yield (Scheme 1). The reaction initiates via NaOH-mediated activation of N-chlorophthalimide (**1-S’**) [38], furnishing intermediates **1-I** and **1-II**. Subsequently, intermediate **1-I** engages in nucleophilic addition with β-nitrostyrene (**1-III**), yielding the chloronium ion intermediate **1-IV**. Under NaOH promotion, intermediate **1-II** selectively attacks the α-position of chloronium intermediate **1-IV**, which bears a higher partial positive charge compared to the β-position, leading to ring-opening and formation of monohaloamino product **1-V**. Finally, the deprotonation/electrophilic chlorination reaction of precursor **1-V** generates the final product **1a** (Scheme 2).

**Scheme 3:**
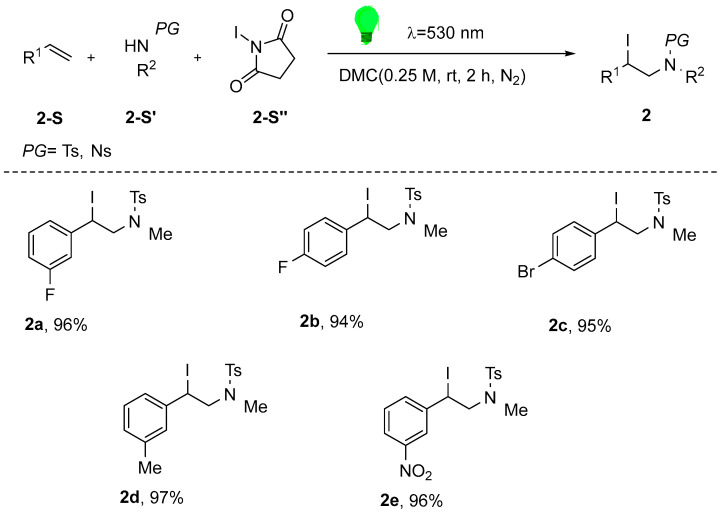
Visible-light-driven, catalyst-free iodoamination of alkenes: green route to nitrogen heterocycles.

In 2021, Engl et al. [39] developed a visible-light-mediated iodoamination protocol for olefins using NIS and readily available sulfonamides as nitrogen sources. The study builds upon their prior expertise in the domain of photocatalytic atom transfer radical addition (ATRA) reactions. This metal-free protocol demonstrates excellent environmental sustainability and high yields using green solvents, without requiring catalysts. Gram-scale synthesis enabled efficient access to bioactive heterocycles—including morpholines, piperidines, and pyrrolidines—from the iodoamination products (Scheme 3).

**Scheme 4:**
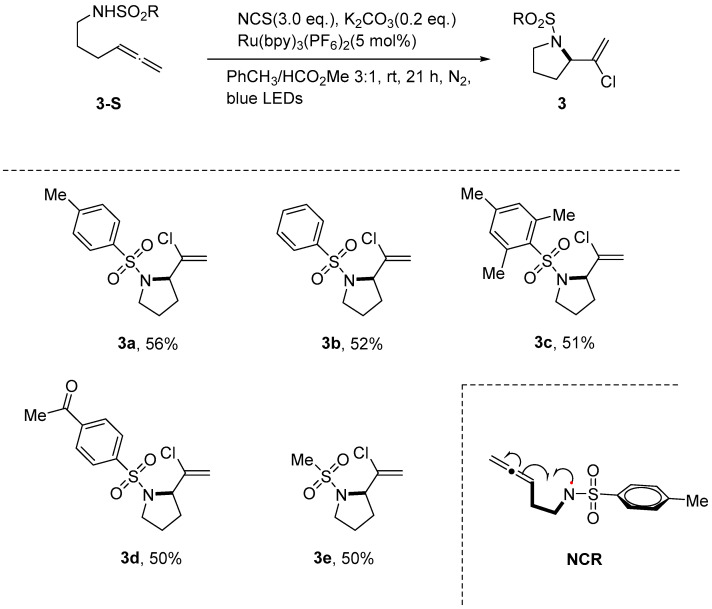
Photoinduced chloroamination cyclization cascade with N-chlorosuccinimide.

Azzi et al. [40] employed N-chlorosuccinimide (NCS) as a chlorine source to synthesize 2-(1-chlorovinyl) pyrrolidines and related heterocycles through photoinduced chloroamination cyclization. The study demonstrates that the critical nitrogen-centered radical (NCR) can be generated either from the photo-excited-state oxidation of Ru-catalyzed deprotonated alkenes or through the photolysis of N-chloroallene. The NCR-induced intramolecular cyclization generates a transient pyrrolidine vinyl radical, which is trapped by NCS to afford the final product. Notably, NCS plays a dual role: activating sulfonamides and delivering chlorine atoms (Scheme 4).

## 3. Intramolecular Stereoselective Haloamination

**Scheme 5:**
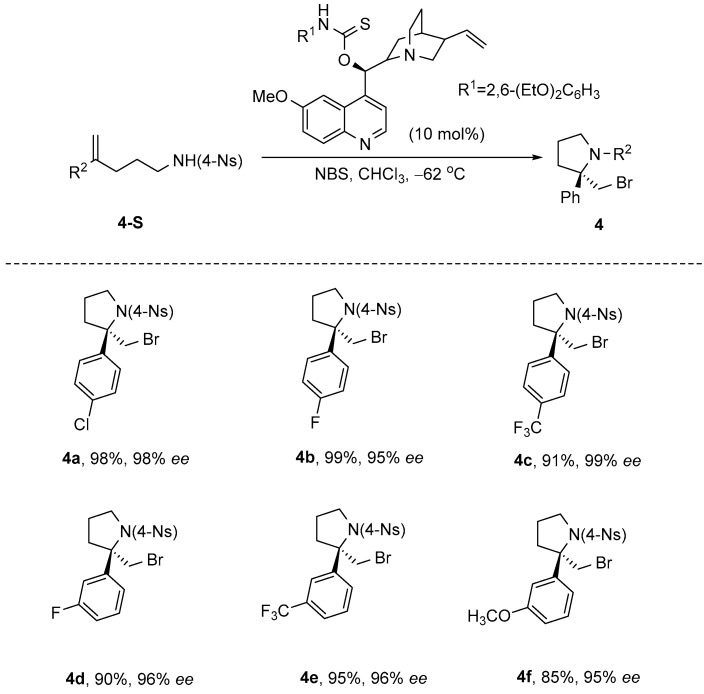
Amino−thiocarbamate catalyzes enantioselective bromoamination cyclization.

In 2011, Zhou et al. [41] reported an efficient and straightforward enantioselective bromoamination cyclization reaction of unsaturated sulfonamides. This reaction employed amino-thiocarbamate as the catalyst, N-bromosuccinimide (NBS) as the brominating agent, and dichloromethane as the solvent, and was conducted at a temperature of −78 °C. The optimized reaction achieved near-quantitative yield (99%) and excellent enantioselectivity (99% *ee*), marking the first catalytic enantioselective halo-N-cyclization reported to date. Furthermore, the tunable aryl thiocarbamate and 6-alkoxyquinoline moieties within the alkaloid framework substantially broaden the substrate scope (Scheme 5).

**Scheme 6:**
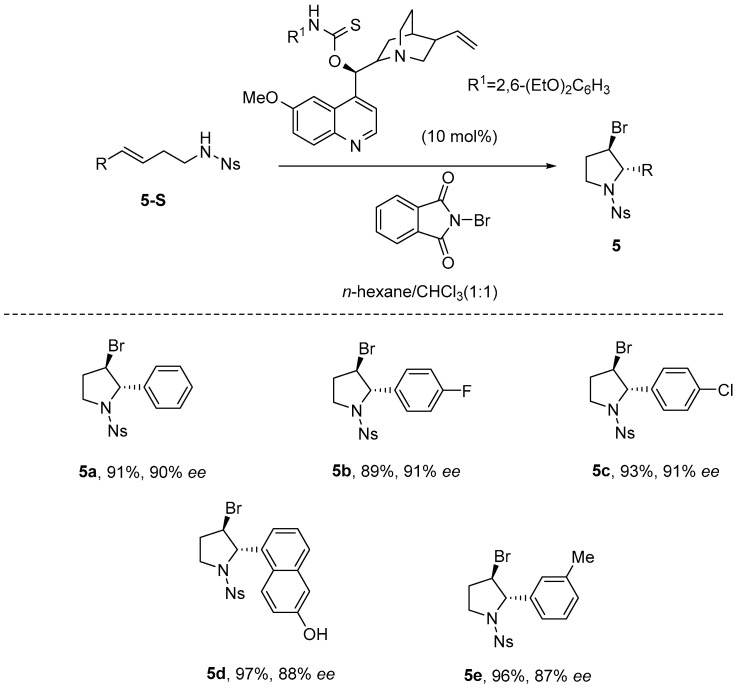
Asymmetric bromoamination catalyzed by amino-thiocarbamate.

The following year, Chen et al. [42] described an efficient and straightforward approach for the synthesis of highly enantioselective 2-substituted 3-bromopyrrolidines. The work demonstrates an amino-thiocarbamate-catalyzed asymmetric bromoamination cyclization of 1,2-disubstituted olefin amides, efficiently constructing nitrogen-containing heterocycles with high enantioselectivity (Scheme 6).

**Scheme 7:**
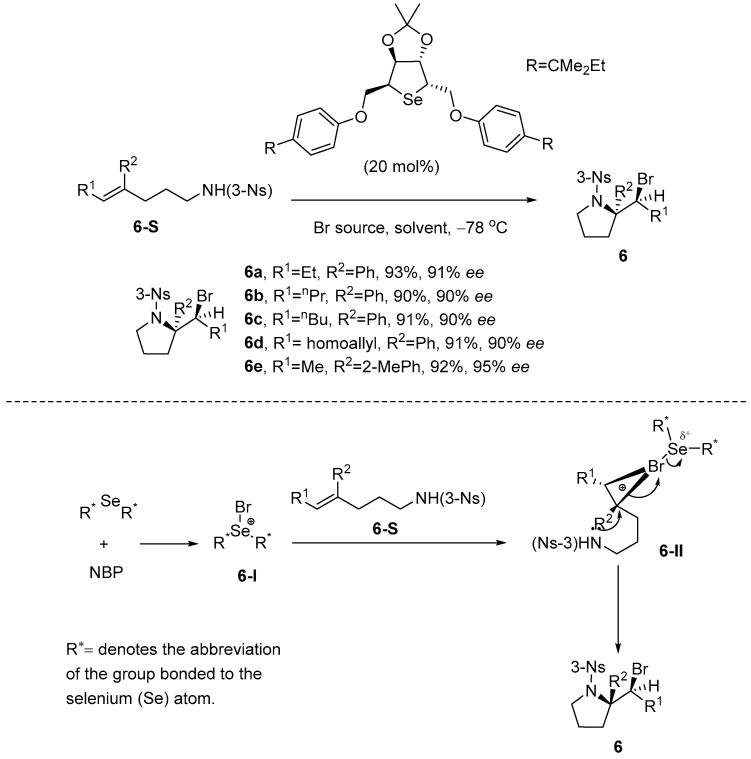
The C_2_-symmetric mannitol-derived cyclic selenocarbamate catalyst can catalyze enantioselective bromoamination cyclization reactions.

Chen and co-workers [43] developed the first Lewis basic selenium-catalyzed asymmetric bromoaminocyclization of trisubstituted olefinic amides, achieving excellent enantioselectivity. Despite having a reaction rate comparable to thiocarbamate, selenocarbamate exhibits instability and is prone to decomposition, presenting challenges in storage. To address this challenge, the research team strategically incorporated selenium atoms into a dialkyl-substituted framework, successfully developing a C_2_-symmetric mannitol-derived cyclic selenocarbamate catalyst for enantioselective N-Bromophthalimide (NBP)-mediated bromoaminocyclization. The monofunctional cyclic selenium catalyst outperformed the bifunctional thiocarbamate catalyst, delivering the enantioenriched pyrrolidine with two stereocenters with higher yield and enantioselectivity. Subsequent rearrangement of this intermediate afforded 2,3-disubstituted piperidines with exceptional enantiocontrol. The reaction mechanism is as follows: First, NBP binds to Lewis basic selenium to generate the electrophilic bromine compound **6-I**. Subsequently, **6-I** binds to the substrate to generate the Se−Br−olefin intermediate **6-II**. Finally, **6-II** is attacked by sulfonamidic S_N_2 in the presence of a catalyst to give the product. The tight pairing structure of intermediate **6-II** is hypothesized to potentially mitigate halogen degradation and racemization (without the use of a bifunctional pocket that could encapsulate the halonium intermediate), which could account for the high enantioselectivity of the product. This C_2_-symmetric cyclic selenium-catalyzed bromoaminocyclization mechanism belongs to the Lewis-base-activated electrophilic haloamination reaction mechanism. The precise stereocontrol of the bromonium ions was realized by the formation of a tight Se-Br coordination intermediate **6-II** between the selenium catalyst and NBP, avoiding the nonselective side reactions commonly found in free radical mechanisms, thus obtaining enantioselectivities of up to 92% *ee*. Compared with the haloamination radical mechanism, this mechanism can be realized under mild conditions (without light or high temperature) and exhibits excellent functional group compatibility for trisubstituted enamide substrates. Meanwhile, the dynamic regulation of the selenium–bromine bond effectively inhibits the racemization problem caused by the radical chain reaction, as well as avoiding the influence of the instability of radical intermediates on the product yield (Scheme 7).

**Scheme 8:**
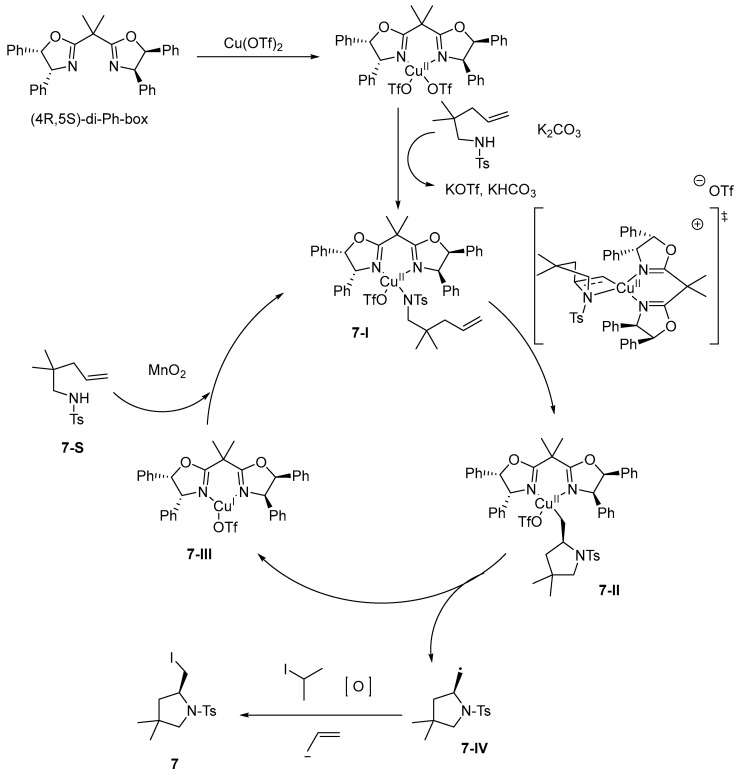
Proposed catalytic cycle of haloamination reaction.

Bovino [44] conducted a thorough investigation into the copper(II)-catalyzed enantioselective haloamination of unactivated olefins, systematically optimizing a range of reaction conditions. This methodology exhibits remarkable versatility in accommodating a wide array of functional groups, resulting in products with moderate to high yields and excellent enantioselectivity. In the catalytic cycle, Cu(OTf)_2_ is initially complexed with the bisoxazoline ligand. Subsequently, the amine is coordinated to the copper center via a base-assisted ligand exchange. This is followed by the formation of a second organocopper complex, wherein the cis-amino group is introduced through a chair transition state, resulting in the formation of a C-N bond. Homocleavage of the C-Cu bond in this complex generates copper(I) and a primary carbon radical. MnO_2_ oxidizes Cu(I) to regenerate the active Cu(II) species, enabling catalytic turnover. The primary carbon radical subsequently reacts with 2-iodopropane to yield an amino halide product and a secondary carbon radical. The secondary radical can react with the primary radical to form an intermolecular carbamylation product, or be oxidized by Cu(II) or MnO_2_ to form the secondary carbocation positive ion and eliminated to form propylene, so that the reaction mixture exists in the form of a gas. This Cu(II)-catalyzed radical-type asymmetric haloamination reaction achieves olefin activation and stereocontrol via Cu(II)–bisoxazoline complexes, combining the high activity of radical reaction with the precision of metal catalysis. Compared with the conventional electrophilic/nucleophilic pathway, the advantages include efficient conversion of unactivated olefins, excellent enantioselectivity via chiral ligands, mild conditions, and compatibility with sensitive functional groups. This synergistic mechanism overcomes both the stoichiometric nature of the radical reaction and the selectivity difficulty of the electrophilic pathway, providing a new approach for complex molecular modification (Scheme 8).

**Scheme 9:**
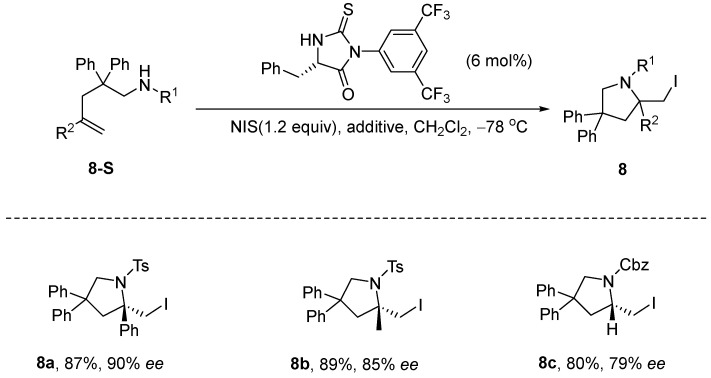
Thiohydantoin catalyzes stereoselective iodoamination of unsaturated olefins.

Mizar et al. [45] reported the development of a novel chiral catalyst for the stereoselective iodoamination of unsaturated olefins. The reaction employed a chiral thiohydantoin catalyst and NIS as the iodine source. Additives (e.g., I_2_, KI, etc.) were found to critically modulate halogen bonding between the catalyst, substrate, and NIS, while substrate electronic effects directly correlated with regioselectivity. While demonstrating promising reactivity, this transformation warrants additional investigation to expand its substrate generality and fully unravel the underlying mechanism (Scheme 9).

**Scheme 10:**
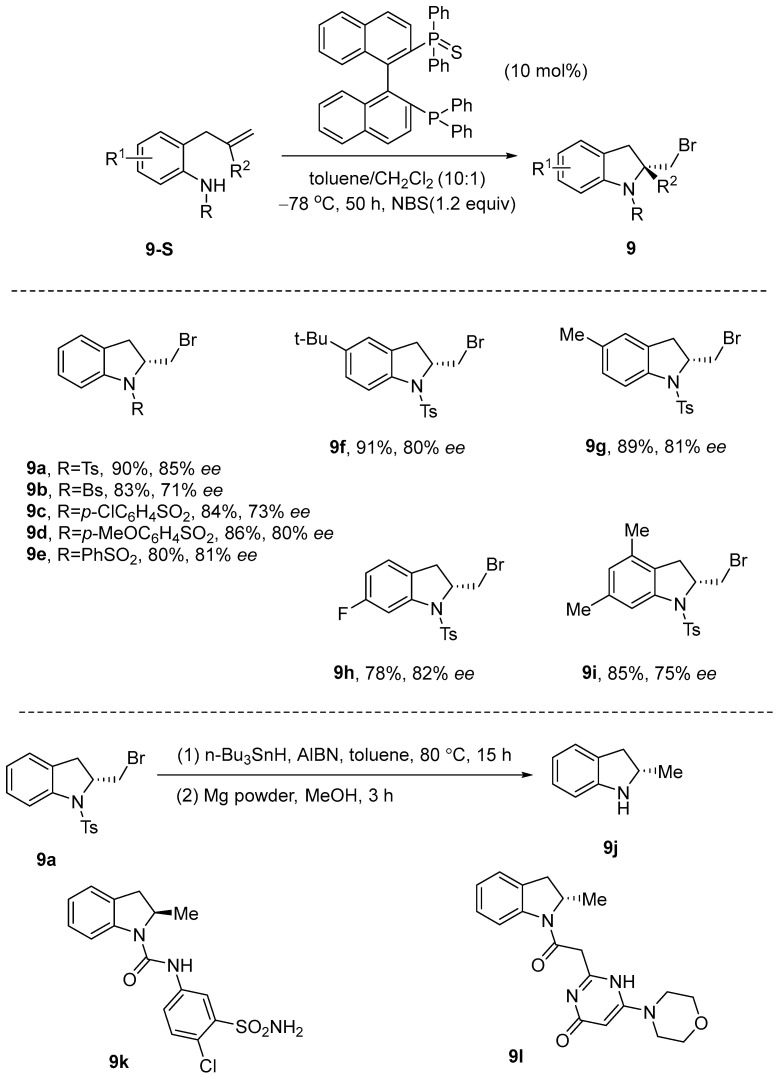
BINAP(S) catalyzes the enantioselective bromoamination of allyl aniline using NBS.

Yu et al. [46] developed a method for the enantioselective bromoamination of allyl anilines utilizing NBS and (S)-2,2′-bis(diphenylphosphino)-1,1′-binaphthyl (BINAP(S)). In this reaction, a series of chiral 2-bromomethyl indolines can be synthesized with high yields and excellent enantioselectivity in a mixed solvent of toluene and dichloromethane (10:1) at −78 °C over a period of 50 h, utilizing BINAP(S) as the catalyst and NBS as the brominating agent. The resulting chiral 2-bromomethyl compounds obtained through this method can be readily transformed into other valuable synthetic intermediates and bioactive compounds. For example, **9a** can undergo a series of reactions to generate the key body **9j**, which can serve as a chiral indole-derived scaffold for the oral antihypertensive diuretic indapamide **9k** [47] and possibly the anticancer drug **9l** [48] (Scheme 10).

**Scheme 11:**
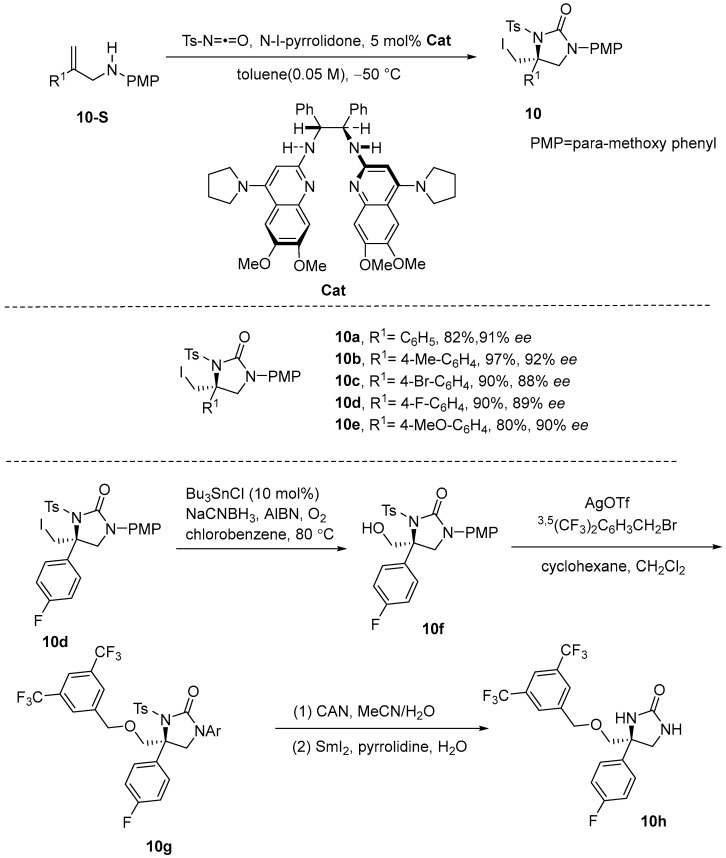
Enantioselective alkene iodoamination for synthesis of chiral cyclic ureas.

Struble et al. [49] developed a carbon–nitrogen symmetric reaction using a C_2_-symmetric BisAmidine (BAM) catalyst, allylic amines, and isocyanates to enrich pairs and prompt conformationally catalyzed iodoamination to produce the urea derivative. In their paper, the catalyst for C_2_-symmetric BisAmidine (BAM) was optimized to mitigate the electronic and spatial deactivation of nitrogen, thereby enhancing N-selectivity from o-sulfonimide intermediates. This optimization achieved a high degree of control over nitrogen cyclization relative to oxygen cyclization, resulting in the formation of cyclic ureas with high yields and excellent enantioselectivity. Chiral five- and six-membered urea derivatives can be readily derivatized into products such as diamines, hydantoins, and fully deprotected imidazolidinones. The final product **10d** can be converted through a series of reactions to the medicinal NK_1_ inhibitor **10h**. It is noteworthy that the solvent was found to have an important influence on the iodoamination of this olefin in this process (Scheme 11).

**Scheme 12:**
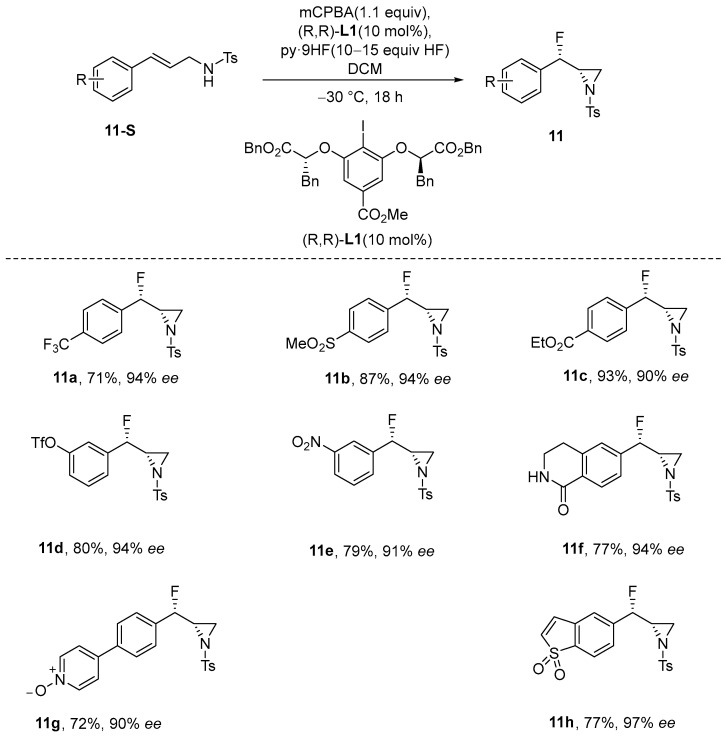
Catalytic enantioselective fluoroamination of alkenes.

Mennie et al. [50] demonstrated the enantioselective fluoroamination of allylamine using a chiral aryl iodide catalyst. This methodology was successfully applied to a diverse range of multifunctional olefinic substrates, utilizing HF-pyridine as a nucleophilic fluorine source and 3-chloroperoxybenzoic acid (mCPBA) as an oxidizing agent. This approach facilitated the synthesis of anti-β-fluoropyrrolidines, along with a variety of 1,2-oxyfluorinated products. This process also allows for the derivation of various α-fluoro-arylethyl amine products, which are obtained in high yields and exhibit excellent enantioselectivities (Scheme 12).

**Scheme 13:**
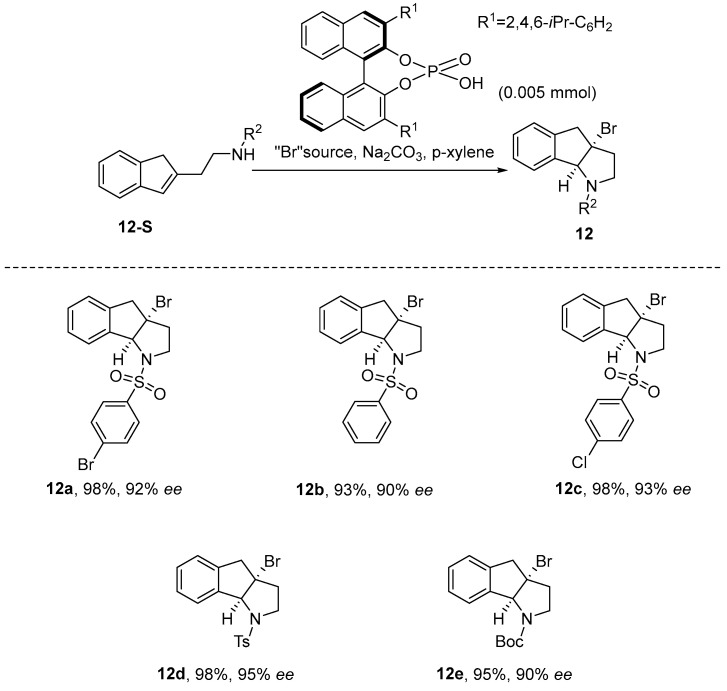
Asymmetric bromoamination cyclization of multifunctional phenylcyclic olefins.

In 2020, Wang et al. [51] introduced an innovative approach for achieving the asymmetric bromoamination cyclization of multifunctional phenylcyclic olefins through a chiral anionic phase-transfer catalysis system. The reaction utilizes a chiral phosphoric acid catalyst, N-Methyl-1,4-diazabicyclo[2.2.2]octanium bromide (DABCO-derived bromide salt) as the bromine source, and p-xylene as the solvent, proceeding under alkaline conditions with sodium carbonate. Under these optimized conditions, the reaction yielded a product with a remarkable 98% yield and an enantiomeric excess (*ee*) of 95%. The results indicate that the reaction is base-constrained, as the active species is a chiral phosphate anion rather than a neutral phosphate. Investigations aimed at expanding the substrate scope revealed that the π-system within the sulfonyl-protecting group of the substrate plays a critical role in influencing enantioselectivity (Scheme 13).

**Scheme 14:**
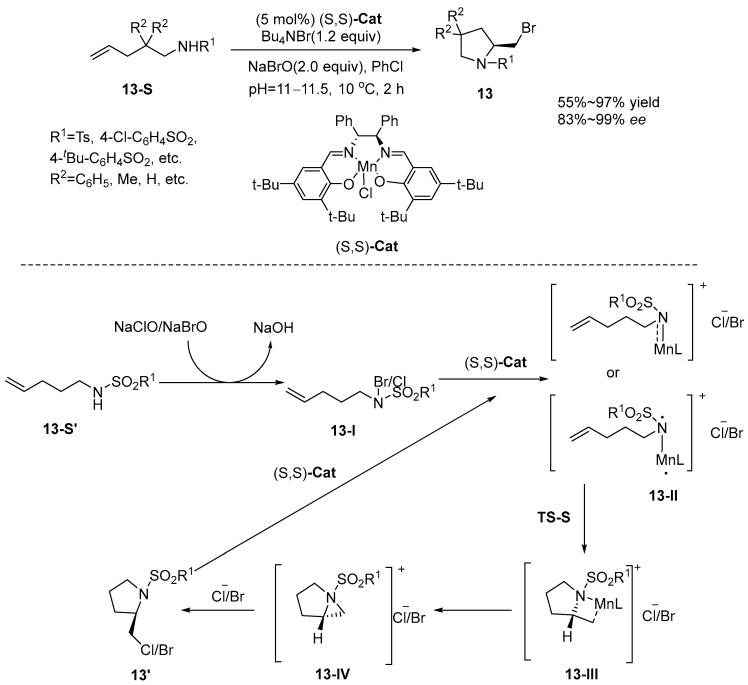
(S,S)-**Cat** enables enantioselective cyclization of olefin haloamination/chiral aziridinium ion intermediate.

Sun et al. [52] conducted a pioneering study that successfully achieved the first enantioselective haloamination reaction through a chiral aziridinium ion intermediate, employing a (S,S)-**Cat** catalyst. Their research presents an innovative approach for the efficient and enantioselective synthesis of intramolecular haloaminated olefins. Specifically, this method facilitates the synthesis of highly enantioselective nitrogen-containing heterocyclic compounds under conditions of pH 11–11.5 and at a temperature of 10 °C, utilizing (S,S)-**Cat** as the catalyst, PhCl as the solvent, and tetrabutylammonium bromide (Bu_4_NBr) as the bromine source. The reaction mechanism is shown in Scheme 14. Initially, the substrate combines with NaClO/NaBrO to form chloramine/bromoamine. In the subsequent step, the chloramine/bromoamine is promptly captured by the catalyst, thereby forming the triplet electronic ground state of the N-Mn intermediate **13-II**. Intermediate **13-II** transforms into intermediate **13-III** via transition-state **TS-R** or **TS-S**. The energy of **TS-S** is calculated to be 4.7 kcal/mol lower than that of **TS-R**. Then intermediate **13-II** undergoes an intramolecular radical addition reaction in the form of **TS-S**, resulting in the formation of chiral intermediate **13-III**. Meanwhile, the site-blocking effect formed by the sulfonyl group in the substrate and the phenyl group in the vicinity of the chiral site in the ligand is not favorable to the formation of **TS-R** configuration. At the same time, the site-blocking effect between the sulfonyl group in the substrate and the phenyl group near the chiral site in the ligand is not favorable to the formation of **TS-R** configuration, while it is favorable to the formation of **TS-S**, which is consistent with the experimental results. Therefore, the desired chiral aziridinium ion intermediate **13-IV** was successfully furnished by (S,S)-**Cat**. Finally, intermediate **13-IV** was attacked by chlorine/bromo ions to obtain the target product. The (Salen)Mn(III)-catalyzed asymmetric intramolecular haloamination reaction, proceeding via a ring-opening pathway of chiral aziridinium ions, offers significant advantages over conventional free radical and electrophilic haloamination mechanisms. Specifically, the rigid structure of the aziridinium intermediate, combined with the ligand-site-blocking effect, enables enantioselectivities as high as 99% *ee*. In contrast, free radical mechanisms are often limited by substrate constraints, while electrophilic haloamination struggles to stabilize chloronium ions. This system is compatible with a broad range of substrates, including electron-rich and electron-deficient olefins as well as heterocycles, under mild conditions, overcoming the limitations of traditional chloroamination methods. The triplet Mn-N intermediate suppresses free radical side reactions via aziridine transfer, while C5-C6 bond polarization governs regioselectivity (rr 6:94). This addresses the long-standing challenge of poor selectivity associated with halonium ions in electrophilic pathways, representing an innovative haloamination mechanism (Scheme 14).

**Scheme 15:**
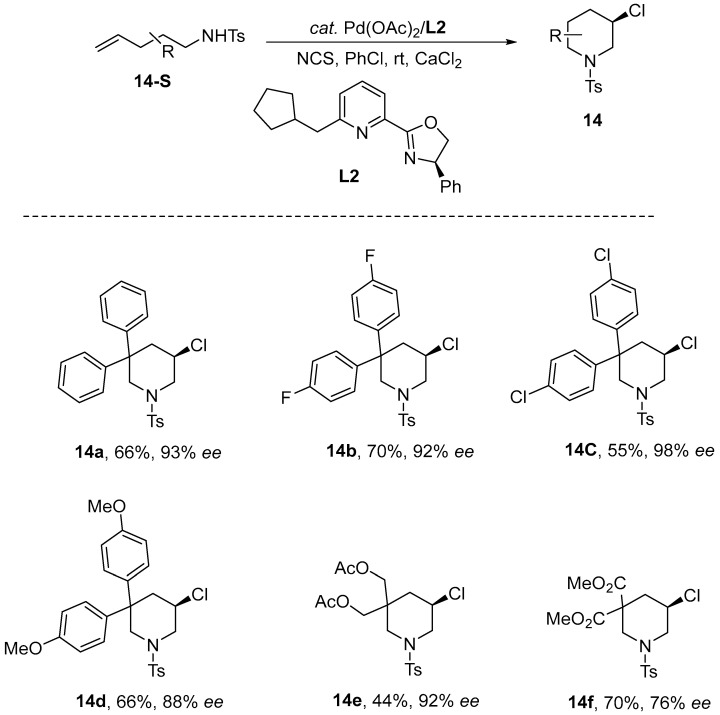
A novel palladium-catalyzed enantioselective chloroamination.

Wang and co-workers disclosed in 2023 a palladium-catalyzed enantioselective 6-endo chloroamination of alkenes [53]. This method enabled the synthesis of variously structured 3-chloropiperidines in high yields (up to 96%) and excellent enantioselectivity (up to 95%). The success of this reaction critically depends on the electrophilic chlorination reagent NCS and a bulky chiral pyridine–oxazoline (Pyox) ligand. Despite these specific requirements, their approach represents a significant advancement in the preparation of chloropiperidines (Scheme 15).

## 4. Intermolecular Stereoselective Haloamination

### 4.1. Intermolecular Diastereoselective Haloamination

**Scheme 16:**
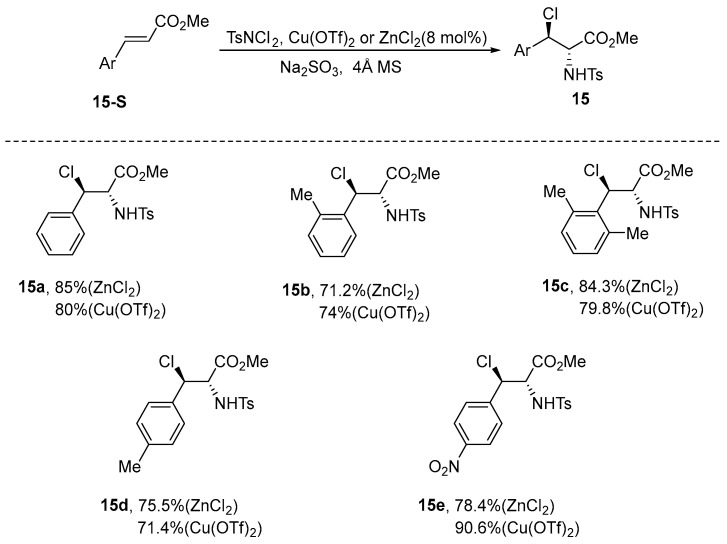
Cu(OTf)_2_/ZnCl_2_-catalyzed diastereoselective chloroamination of cinnamic esters.

Cinnamic esters are widely recognized as versatile substrates in synthetic chemistry, particularly in the oxidation of olefins [54]. These cinnamic esters have been effectively utilized in various reactions, including catalyzed asymmetric dihydroxylation [55,56], epoxidation [57], aziridination [58,59], and aminohydroxylation reactions [60,61]. Such transformations provide a robust and effective strategy for the synthesis of complex molecular architectures. Building on the foundation of cinnamon esters, Li et al. developed an innovative method for the synthesis of adjacent haloamine derivatives. This process is carried out in acetonitrile as the solvent, with zinc chloride or Cu(OTf)_2_ serving as catalysts. N,N-dichloro-p-toluenesulfonamide functions as both the chlorine and nitrogen source for the reaction. The experimental findings reveal that this synthetic method not only yields products in good to excellent quantities, but also displays advantageous stereoselectivity. Notably, for two isomers that were challenging to separate via column chromatography, the trans/cis ratios were 4/1 when using ZnCl_2_ and 5/1 when employing Cu(OTf)_2_ [62] (Scheme 16).

**Scheme 17:**
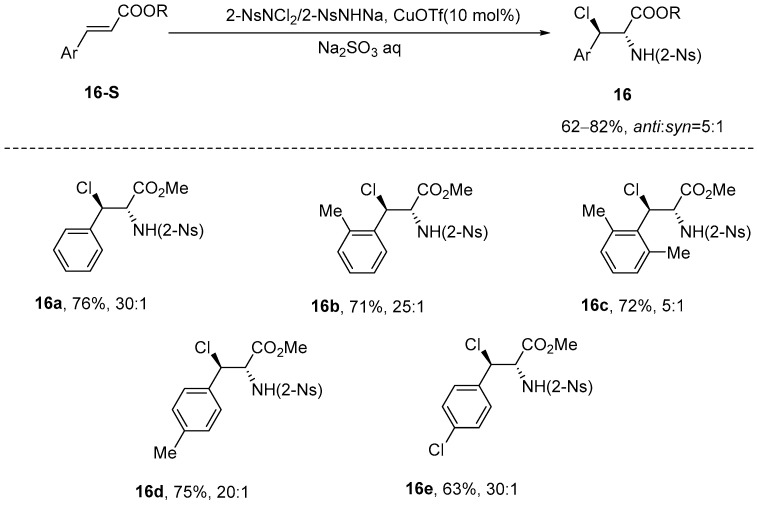
CuOTf-catalyzed chloroamination of cinnamic esters.

In 2000, Li et al. [63] introduced a novel method for the synthesis of anti-alkyl 3-chloro-2-(o-nitrobenzenesulfonamido)-3-phenylpropionate derivatives. The synthesis involved the diastereoselective chloroamination of cinnamate using 2-NsNCl_2_/2-NsNHNa as the nitrogen and chlorine sources, with CuOTf serving as the catalyst. This methodology yielded the target compound with high efficiency and exceptional diastereoselectivity. Furthermore, the amine was protected with an N-(p-nitrobenzenesulfonyl) (N-nosyl) group, which can be easily removed to facilitate subsequent derivatization. Additionally, the intermediate N-nosyl-N-chlorocyclopropylamine generated during the synthesis demonstrates considerable potential for application in organic nucleophilic reactions (Scheme 17).

**Scheme 18:**
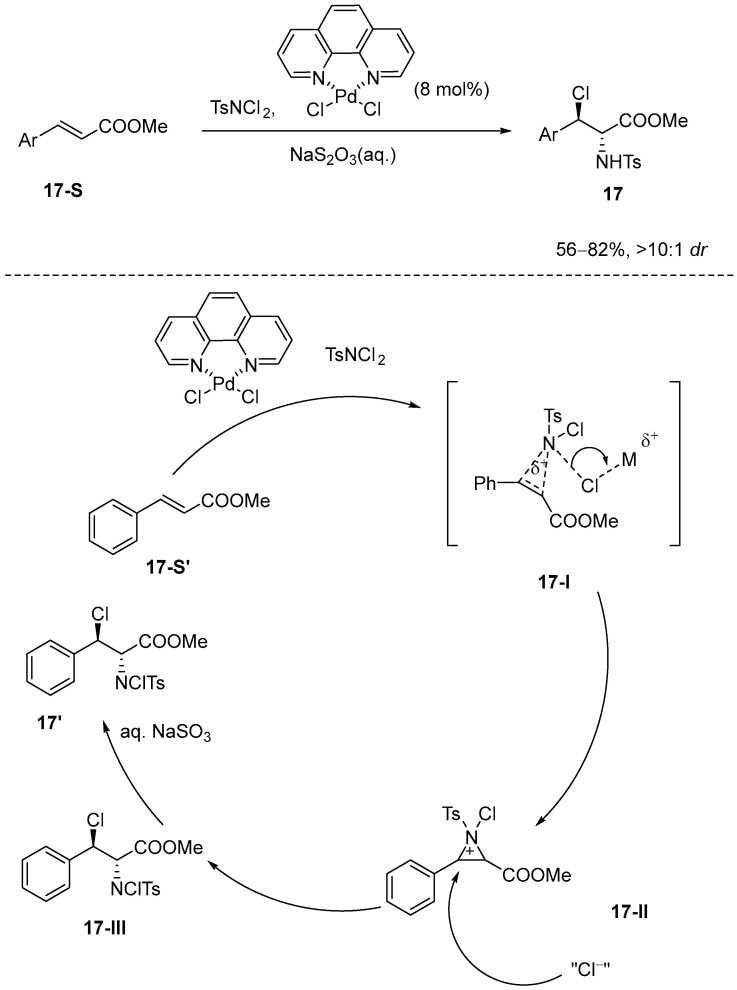
Transition-metal-catalyzed diastereoselective haloamination of cinnamic esters.

Wei et al. [64] were the first to report an diastereoselective haloamination reaction of cinnamic esters utilizing palladium catalysis. In their study, N-Chloro-p-toluenesulfonamide (p-TsNCl) was utilized as both the nitrogen and chlorine source, while dichloro-(1,10-phenanthroline)-palladium(II) was employed as the catalyst to facilitate the haloamination process. The results indicated that the yield of the resultant product ranged from 56% to 82%, accompanied by exceptional stereoselectivity, exceeding 10:1. Furthermore, Wei’s group proposed an innovative mechanism in which olefins undergo electrophilic amination to generate an N-tosyl N-chloro aziridinium intermediate. This intermediate demonstrates reactivity toward a diverse array of nucleophiles and possesses significant potential for extensive applications in organic synthesis (Scheme 18). Wei et al. proposed a novel reaction mechanism, as illustrated in Scheme 18, which suggests the formation of a new intermediate, specifically the N-tosyl N-chloro intermediate. Initially, the palladium metal center of the catalyst selectively coordinates with the N-Cl group rather than the oxygen atoms in N,N-dichloro-p-toluenesulfonamide. This selective coordination is essential for forming the ‘Ns-N^+^-Cl’ structure, which is necessary for subsequent electrophilic additions. Subsequently, the chlorine ligand coordinated to palladium acts as a nucleophile, attacking the three-membered ring of the N-tosyl N-chloro aziridinium ion. This attack results in ring-opening and the formation of the final product through an S_N_2 mechanism.

**Scheme 19:**
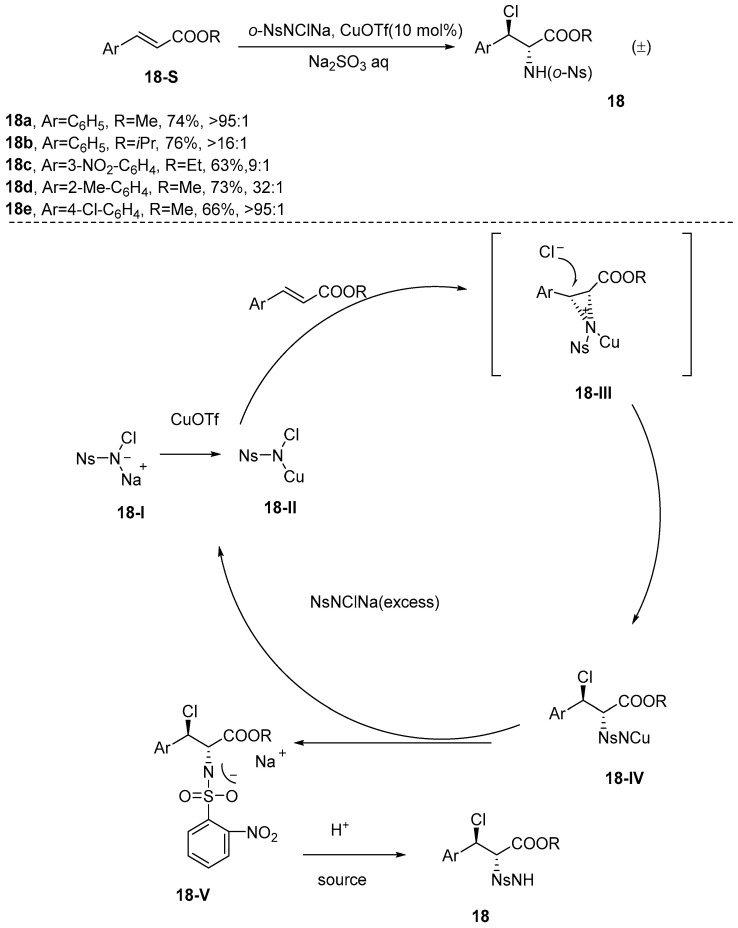
The diastereoselective chloroamination reaction utilizing N-chloro-N-sodium sulfonamide as the nitrogen and chlorine source.

In 2001, Li et al. [65] demonstrated that the use of N-chloro-N-sodium-sulfonamide as a source of nitrogen and chlorine in haloamination reactions provides enhanced operational simplicity compared to the previously reported mixtures of NsNCl_2_ and NsNHNa. Furthermore, in the presence of a copper catalyst, the reaction between N-chloro-N-sodium-sulfonamide and unsaturated olefins did not yield the anticipated aziridine; instead, o-haloamide derivatives were obtained. This phenomenon is attributed to the formation of unprecedented N-chloro-N-copper-2-nitrobenzenesulfonyl aziridinium intermediates in the reaction, which significantly enhances the asymmetry of the reaction. Consequently, the resulting products demonstrate excellent stereoselectivity, exceeding a ratio of 95:1 (Scheme 19). The reaction mechanism depicted in Scheme 19 unfolds as follows: Initially, compound **18-I** undergoes a reaction with copper(I) triflate, resulting in the formation of N-chloro-N-copper-2-nitrobenzenesulfonamide, designated as compound **18-II**. This intermediate subsequently undergoes a distinctive transformation through its reaction with an olefin substrate, leading to the formation of an unprecedented N-chloro-N-copper-aziridinium intermediate, designated as compound **18-III**. In this compound, copper is either coordinated to the nitrogen atom or exists in proximity to the sulfonyl oxygen. Finally, the chloride ion (Cl^−^) attacks the carbocationic active site of intermediate **18-III** through an S_N_2 mechanism. Given that the β-position exhibits a greater positive charge compared to the α-position, it is crucial to introduce an excess of 2-NsNClNa to promote the formation of intermediate **18-V**. Subsequently, N-chloro-N-copper-2-nitrobenzenesulfonamide (compound **18-II**) can be regenerated from intermediate **18-V**, thereby facilitating cyclic catalysis.

**Scheme 20:**
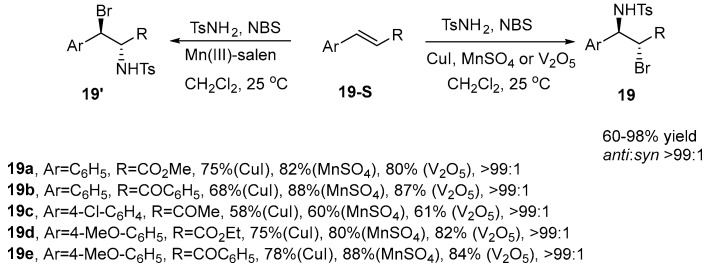
CuI, MnSO_4_, or V_2_O_5_ catalyze the bromoamination of a variety of olefins.

In 2003, Thakur et al. [66] presented a novel methodology for the synthesis of 1,2-bromoaminated compounds. Their approach employs NBS as the brominating agent, p-Toluenesulfonamide (TsNH_2_) as the nitrogen source, and cuprous iodide, manganese(II) sulfate, or vanadium pentoxide as catalysts to promote the bromoamination of a variety of olefins. This method produces products with remarkable stereoselectivity, achieving ratios exceeding 99:1 (Scheme 20).

**Scheme 21:**
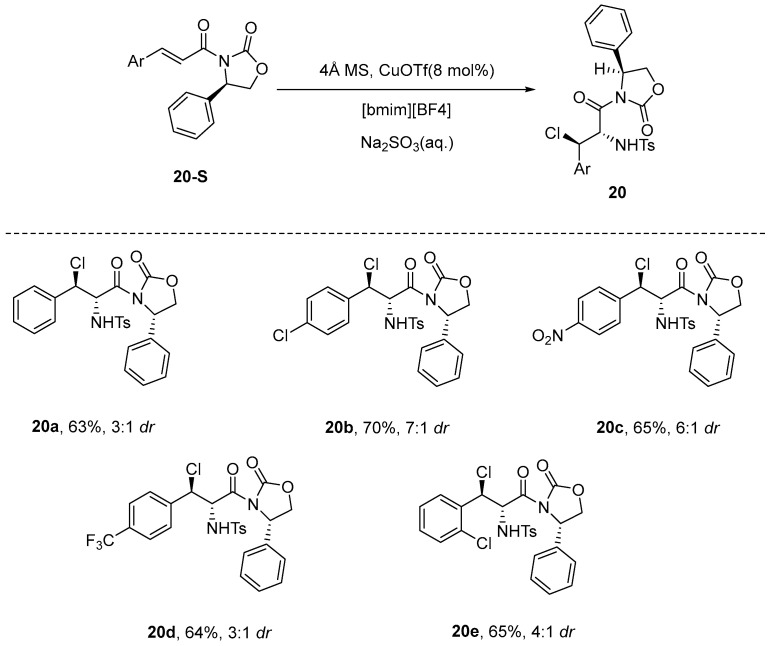
Ionic-liquid-mediated diastereoselective haloamination of olefins.

In 2004, Xu et al. [67] were the first to report the utilization of ionic liquids as mediators for the asymmetric haloamination of olefins. When α,β-unsaturated N-acyl-4-alkyloxazolidinones were employed as substrates for this reaction, conventional organic solvents proved inadequate in facilitating the reaction under standard conditions. In contrast, the ionic liquid 1-butyl-3-methylimidazolium tetrafluoroborate ([bmim][BF_4_]) facilitated the reaction efficiently, resulting in products with high chemical yields and excellent enantioselectivity. Additionally, [bmim][BF_4_] possesses several advantageous properties: it is non-volatile and non-flammable, exhibits high solubility for polar compounds, is readily recyclable, and offers green chemistry advantages (Scheme 21).

**Scheme 22:**
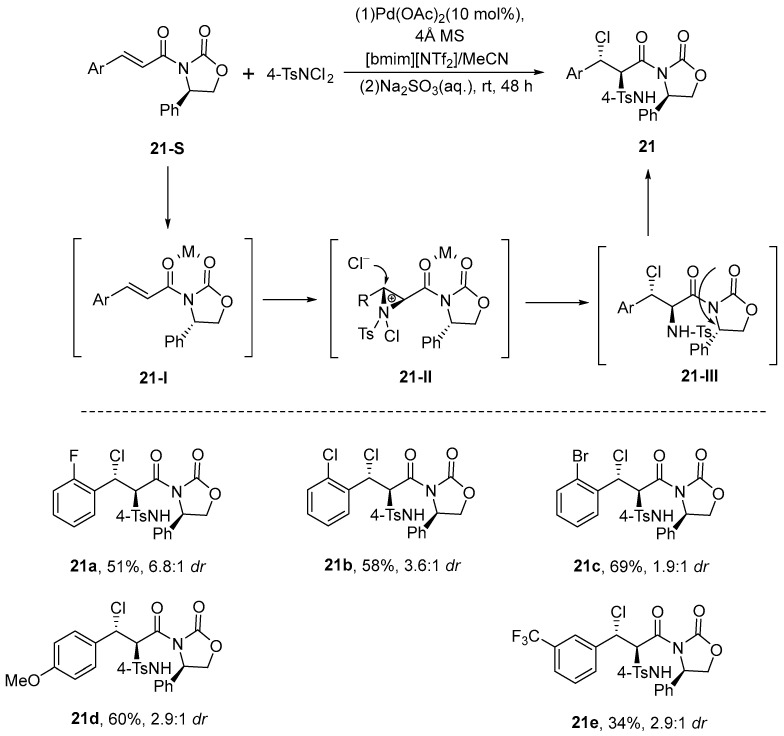
Chelation-controlled diastereoselective haloamination.

In 2007, Wang et al. [68] reported a chelation-controlled asymmetric haloamination reaction using Pd(OAc)_2_ as the catalyst and acetonitrile (MeCN) as the solvent. This reaction was conducted in the ionic liquid 1-butyl-3-methylimidazolium bis(trifluoromethanesulfonyl)imide ([bmim][NTf_2_]), a medium extensively studied in prior works [69,70,71,72]. This reaction can be conducted in a single pot without the requirement for inert gas protection. The method yields moderate to excellent results with respect to both yield and diastereoselectivity. The possible mechanism of this reaction is as shown in the figure, where the nitrogen electrophile attacks the carbon–carbon double bond on the side with less steric hindrance, and the Cl^−^ ion attacks the other side of the double bond. Among them, the Pd metal acts as the coordination center to form a chelating complex (Scheme 22).

**Scheme 23:**
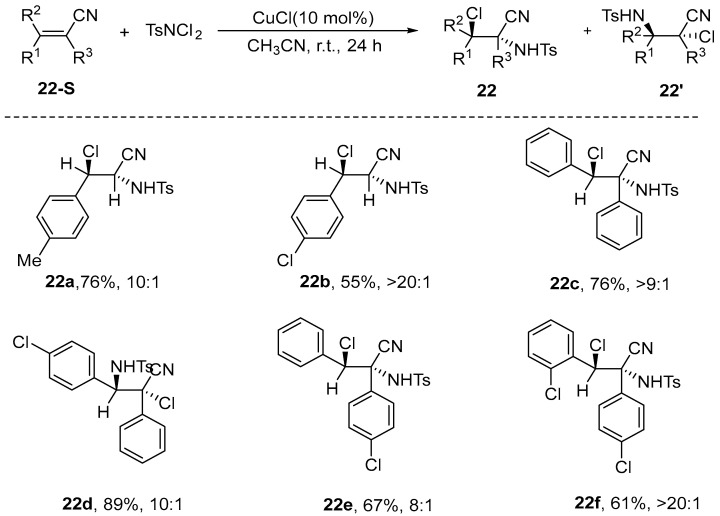
Haloamination of 3,3-diphenylacrylonitrile.

Han et al. [73] recently introduced an innovative asymmetric haloamination method that employs α,β-unsaturated nitriles as the substrate for the reaction. This approach represents a departure from earlier methodologies that primarily utilized α,β-unsaturated ketones, esters, methylcyclopropanes, or vinyl cyclopropanes as substrates. In this methodology, cuprous chloride is employed as a catalyst at a molar ratio of 10 mol%, alongside N-dichloro-p-toluenesulfonamide (4-TsNCl_2_) and α,β-unsaturated nitrile serving as substrates, with acetonitrile utilized as the solvent. The reaction is carried out in the presence of 4 Å MS. Notably, this method does not require inert gas protection and proceeds efficiently at room temperature over a period of 24 h, yielding a diverse array of halogenated amino nitriles with high yields, exceptional enantioselectivity. This innovative method offers a convenient approach for synthesizing a broad spectrum of adjacent haloamination nitrile compounds, while simultaneously broadening the applicability of existing haloamination substrates (Scheme 23).

**Scheme 24:**
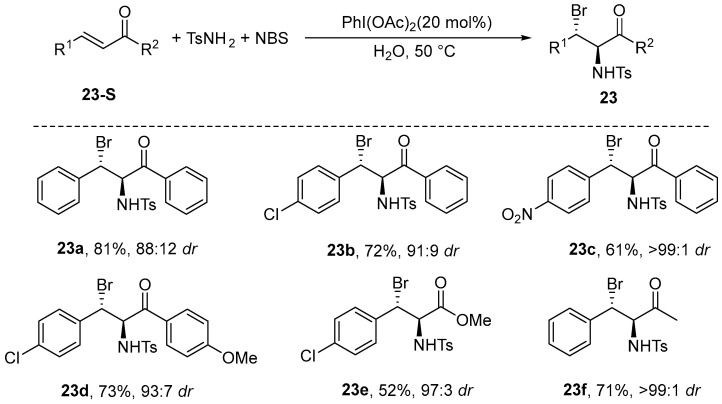
PhI(OAc)_2_ catalyzes the bromoamination of electron-deficient olefins in pure water.

In 2009, Wu et al. [74] reported the development of an asymmetric haloamination reaction involving electron-deficient olefins, which was conducted in pure water. The reaction was conducted under pure aqueous conditions, utilizing TsNH_2_ and NBS as the sources of nitrogen and bromine, respectively, with PhI(OAc)_2_ acting as the catalyst. This process yielded products with high efficiency, as well as excellent diastereoselectivity. The methodology demonstrates broad applicability to a diverse array of olefins, including α,β-unsaturated ketones, styrene, cinnamic esters, and other cinnamyl-derived olefinic compounds, while maintaining exceptional diastereoselectivity (Scheme 24).

**Scheme 25:**
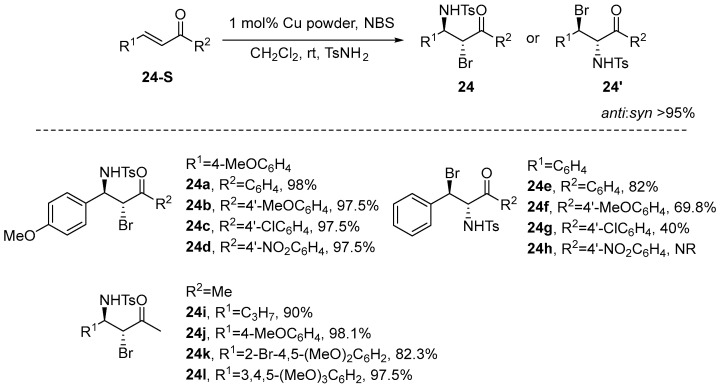
Copper powder mediates stereoselective bromoamination of α,β-unsaturated ketones.

Shi et al. [75] proposed a reaction that utilizes copper powder as a catalyst for the regio- and stereoselective haloamination of β-unsaturated ketones. This method employs 4-Toluenesulfonamide (4-TsNH_2_) as the nitrogen source and NBS as the bromine source, resulting in products with exceptionally high yields and excellent regio- and stereoselectivity. Notably, the ratio of anti to syn products exceeds 95%, indicating that no syn isomer was detected by 1 H NMR. Furthermore, the study demonstrated that the strong electron-donating substituents significantly enhance the reactivity of the double bond in α,β-unsaturated ketones toward 1,4-addition with electrophilic brominating reagents (NBS) by increasing the electron density of the double bond. Conversely, the observed suppression of the addition reaction by strong electron-withdrawing groups provides direct evidence for the electrophilic nature of this transformation, as these substituents decrease the electron density available for attack by NBS (Scheme 25).

**Scheme 26:**
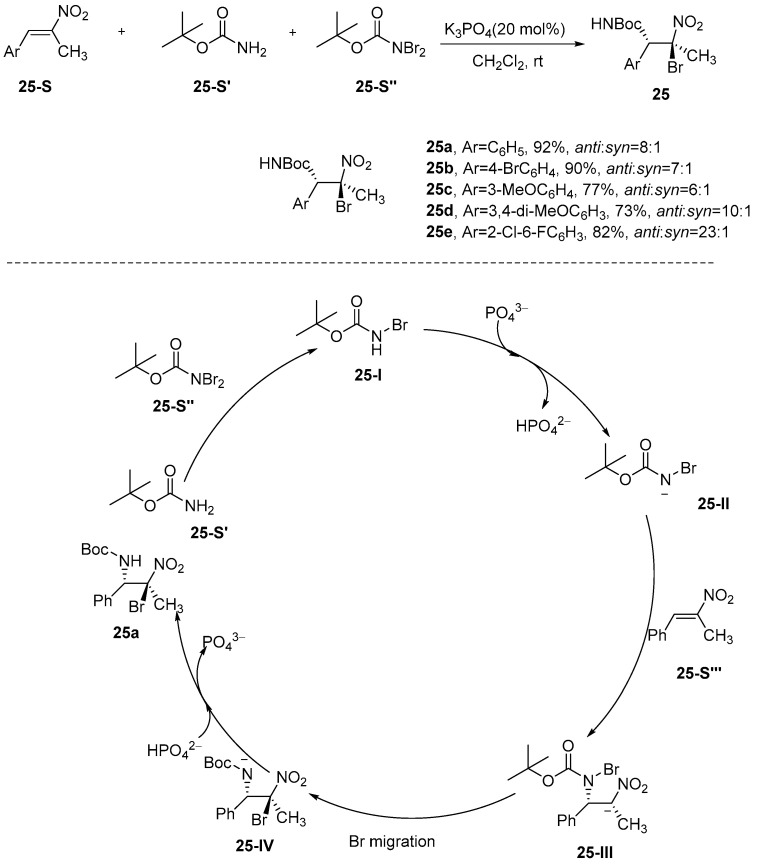
A stereoselective bromoamination reaction.

Chen et al. [76] reported a stereoselective bromoamination reaction utilizing α,β-unsaturated nitro compounds in conjunction with t-butyl N,N-dibromocarbamate and t-butyl carbamate as the bromine and nitrogen sources, respectively, with K_3_PO_4_ serving as the catalyst. The catalytic system exhibits remarkable versatility in accommodating a wide range of β-methyl-β-nitrostyrene substrates, achieving excellent chemical yields along with superior stereoselectivity. Furthermore, the N-protecting group can be efficiently removed under mild conditions, resulting in the formation of the corresponding free bromide product (Scheme 26). The reaction mechanism, as illustrated in Scheme 26, proceeds as follows: Initially, N,N-Dibromo-tert-butoxycarbamide (BocNBr_2_) reacts with tert-Butoxycarbonylamine (BocNH_2_) to form intermediate **25-I**. Subsequently, intermediate **25-I** is deprotonated by K_3_PO_4_, resulting in the formation of intermediate **25-II**. Finally, intermediate **25-II** undergoes a Michael addition reaction with the substrate, leading to the formation of intermediate **25-II**. The bromide ions subsequently migrate from the N-Br position in the amide to the site of negative charge, resulting in the formation of intermediate **25-IV**. Intermediate **25-IV** is then protonated by HPO_4_^2−^ to yield product **25a**, simultaneously regenerating the catalyst PO_4_^3−^. The reaction belongs to the nucleophilic haloamination mechanism, and achieves high selectivity through the synergistic pathway of nitrogen-negative ion Michael addition and bromine migration, which is innovative in the following ways:

The strong electron-deficient nitroolefin is utilized to activate the β-site to achieve the nitrogen-negative ion direct addition; the bromine migration mechanism replaces the traditional electrophilic haloamination, avoiding the instability of halonium ions; and it provides a transition-metal-free, mild, and highly efficient new strategy for the synthesis of vicinal bromoamine. 

**Scheme 27:**
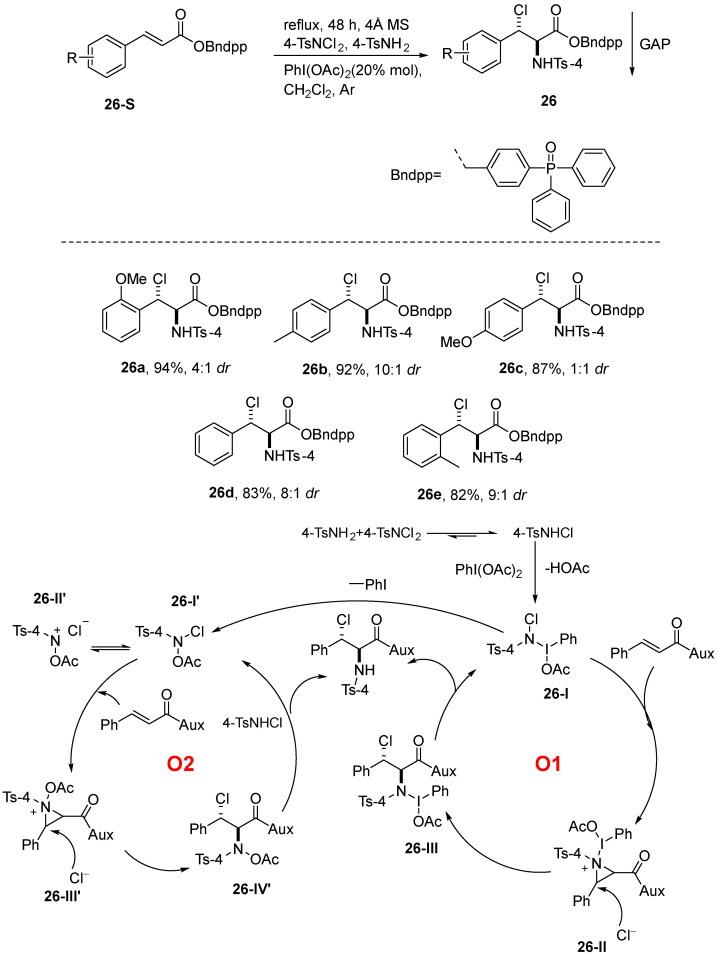
The GAP method of high-valent iodine(III) used for diastereoselective chloroamination.

Rahman et al. [77] introduced a Group-Assisted Purification (GAP) method that utilizes a high-valent iodine(III)-directed functional group to purify olefins via an asymmetric chloroamination reaction. In this methodology, dppBnOH and dppBnNH_2_ (where dpp denotes diphenylphosphine oxide) were utilized as substrates, while 4-TsNH_2_ and N,N-Dichloro-4-toluenesulfonamide (4-TsNCl_2_) served as the sources of nitrogen and chlorine, respectively. PhI(OAc)_2_ was utilized as the catalyst, and methylene chloride functioned as the solvent. The reaction yielded products in moderate to good yields, demonstrating excellent regio- and stereoselectivity. The GAP method features operational simplicity, cost-effectiveness, enhanced environmental sustainability, and eco-friendliness. This method is particularly well-suited for the synthesis of various electron-deficient olefins and o-haloamines. The reaction mechanism is that 4-TsNCl_2_ reacts with 4-TsNH_2_ to form N-chloro-p-toluenesulfonamide (4-TsNHCl), a product that may be oxidized by PhI(OAc)_2_ to form intermediate **26-I** following either the **O1** cycle or the **O2** cycle. In the **O1** cycle, intermediate **26-I** is conjugated with the substrate to form nitrogen–iridium **26-II**, which is subsequently attacked by the anionic attack of the dissociated intermediate **26-II** to generate **26-III**. The nitrogen–iridium intermediate **26-II** formed in the **O1** cycle ensures subsequent anionic attack occurs at specific spatial sites through the directing effect of coordination bonds, thereby achieving excellent stereoselectivity. **26-III** in combination with 4-TsNHCl generates the final product. Upon release due to dissociation of the unstable N-I bond of intermediate **26-I**, it is possible to form the reactive intermediate N-acetoxy-N-halo-p-toluenesulfonamide **26-I’** that follows the **O2** cycle. The intermediate **26-I’** may also be present in the form of **26-II’**, which binds to the substrate to produce the aziridium intermediate **26-III’**, and similarly, **26-III’** is attacked by the anion to produce **26-IV’**, which, in combination with 4-TsNHCl, produces the target product. Furthermore, this reaction achieves excellent regiocontrol through two competitive reaction pathways (**O1** and **O2** cycles) based on a high-valent iodine(III) catalytic system. Particularly noteworthy is that when the substrate is an α,β-unsaturated carbonyl compound, the strong electron-withdrawing effect of the conjugated carbonyl group renders the β-position carbon atom significantly electrophilic, thus preferentially accepting nucleophilic attack from the nitrogen source. Simultaneously, the iodine(III) catalyst precisely directs the selective addition of halogen at the α-position through the formation of a crucial nitrogen–iodine bond intermediate, leveraging its unique steric effects to efficiently construct α-halo-β-amine products. This distinctive “α-halo/β-amine” regioselectivity pattern fully demonstrates the precise control capability of high-valent iodine reagents over reaction sites: the **O1** cycle preferentially achieves β-position amination through a classical ionic reaction mechanism, while the **O2** cycle promotes α-position halogen functionalization via highly reactive nitrene intermediates (Scheme 27).

**Scheme 28:**
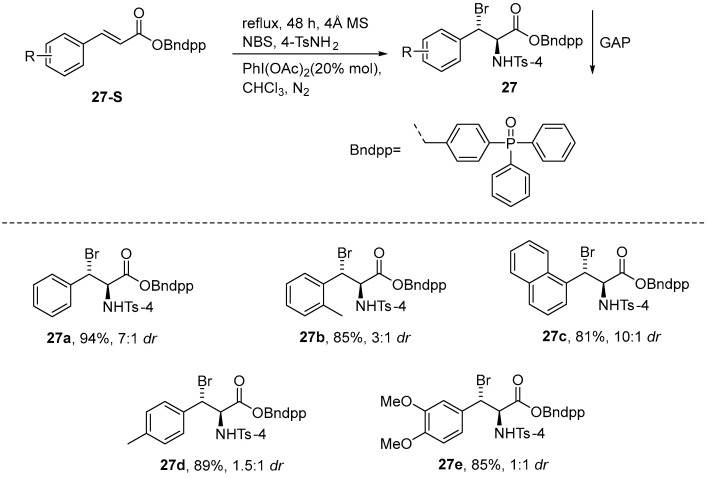
The GAP method of high-valent iodine(III) used for diastereoselective bromoamination.

A year later, Rahman et al. [78] employed the GAP method once again to mediate the diastereoselective bromoamination of electron-deficient olefins using high-valent iodine(III). The method utilizes PhI(OAc)_2_ as a catalyst, along with a GAP-anchored substrate mixed with TsNH_2_-NBS, to produce a diverse array of o-bromamines. The resulting products do not require recrystallization or column chromatography; they only need to be washed with co-solvents, thereby conserving silica, solvents, time, and labor. Furthermore, it is clearly visible from the Scheme that GAP is both recyclable and reusable (Scheme 28).

**Scheme 29:**
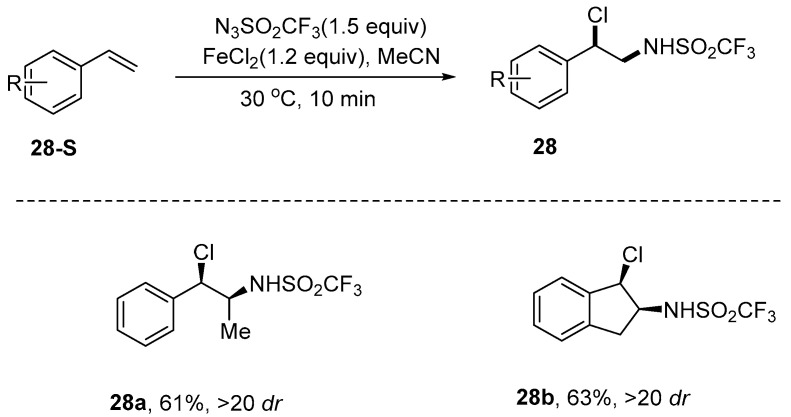
Chloroamination of styrene.

Zhao and his team [79] introduced an innovative approach to achieve a highly efficient chloroamination reaction on p-xylene. In this method, Trifluoromethanesulfonyl azide (N_3_SO_2_CF_3_) served as the aminating agent, ferrous chloride acted as the chlorinating reagent, and acetonitrile was employed as the solvent. This process enabled the efficient synthesis of chloroaminated products with high regioselectivity at a moderate temperature of 30 °C. This approach is distinguished by its operational simplicity, mild reaction conditions, and excellent compatibility with a variety of functional groups. Furthermore, this method can be effectively employed to facilitate bromoamination reactions. Additionally, the incorporation of sodium azide allows for a one-pot aminoazide reaction of styrene within the same reaction system. This reaction system achieves regiocontrol through a radical mechanism. In the case of p-xylene substrates, the relatively low homolytic cleavage energy of benzylic C-H bonds enables the N_3_SO_2_CF_3_/FeCl_2_ system to preferentially initiate chloroamination at this position. For α,β-unsaturated carbonyl compounds, the strongly electron-withdrawing sulfonamido group (-SO_2_CF_3_) directs chlorine radicals to selectively attack the electron-deficient β-position through polarization effects, while nitrogen radicals add to the relatively electron-rich α-position, ultimately yielding β-chloro-α-amine products. This completely inverted regioselectivity pattern (compared to iodine-catalyzed systems) profoundly demonstrates the decisive influence of reaction mechanisms on selectivity control: in radical pathways, the stability of intermediates serves as the key determinant of regioselectivity. Notably, the regioselectivity in this radical system is primarily governed by the synergistic interplay between substrate electronic effects and radical stability (Scheme 29).

**Scheme 30:**
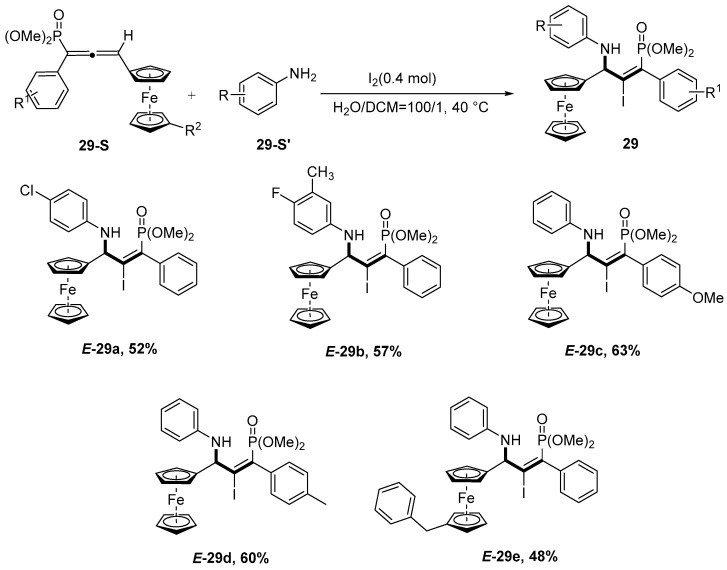
A novel unconventional redox-neutral diastereoselective iodoimination reaction of allenamides.

In 2024, Guo et al. [80] developed a highly regio- and diastereoselective iodoamination of ferrocene-containing allenylphosphonates with anilines, enabling the synthesis of multifunctional tetrasubstituted allylic amines in moderate yields. Notably, this practical protocol simultaneously installs a tetrasubstituted olefin moiety, an iodine atom, a phosphonate group, and a ferrocenyl unit into the allylic amine motif, demonstrating broad substrate scope (45 examples) and scalability (gram-scale synthesis) (Scheme 30).

### 4.2. Intermolecular Enantioselective Haloamination

**Scheme 31:**
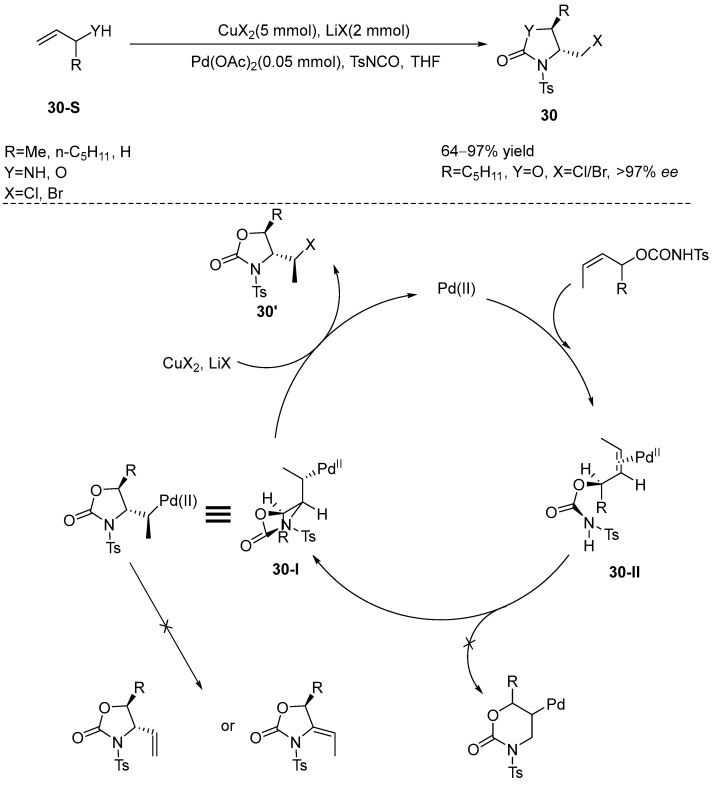
Pd(II) catalyzes the asymmetric haloamination of allyl alcohols or allyl amines.

Lei et al. [81] reported a novel haloamination reaction catalyzed by divalent palladium, which demonstrated high stereoselectivity. The substrates for this reaction encompass allyl alcohols and allyl amines, utilizing Pd(OAc)_2_ as the catalyst, p-toluenesulfonyl isocyanate (TsNCO) as the nitrogen source, copper and lithium halides as halide sources, and tetrahydrofuran (THF) as the solvent. Notably, the reaction initiates with the binding of the substrate to TsNCO, resulting in the formation of the corresponding haloamination products, which are subsequently catalyzed by Pd(II). The proposed mechanism of the reaction is as follows: Initially, the formation of C–Pd bonds occurs via Pd(II)-catalyzed trans-nucleopalladation of the substrate olefin molecule. Subsequently, the oxidative cleavage of the C–Pd bonds is facilitated by the synergistic action of copper halide and lithium halide, leading to the formation of the final product. During this process, only intermediate **30-I** is generated. However, when the substrate features a substituent at the allyl position, intermediate **30-II** is formed instead. The cleavage of the C-Pd bond in this reaction was highly selective, and no other elimination products were observed (Scheme 31).

**Scheme 32:**
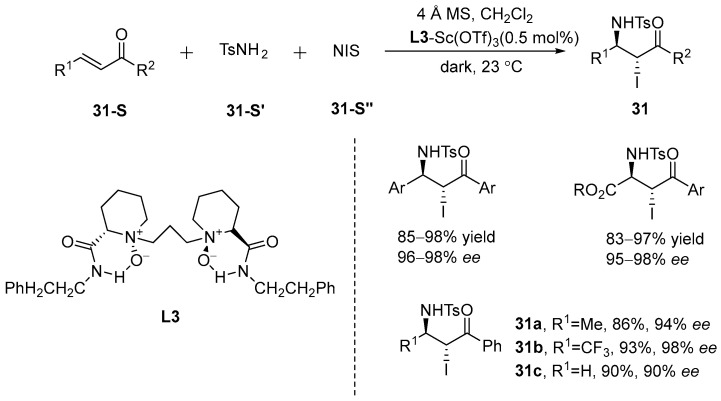
Asymmetric iodoamination reaction of chalcone and 4-aryl-4-oxobutyl-2-enoate as substrates.

Cai et al. [82] identified an asymmetric iodoamination reaction using chalcone and 4-aryl-4-oxobutyl-2-enoate as substrates, achieving remarkable results. The procedure was carried out under anhydrous conditions using freshly dried zeolite (4 Å MS) in the absence of light, with **L3**-Sc(OTf)_3_ as the catalyst and NIS and TsNH_2_ as the iodine and nitrogen sources, respectively, resulting in a product with high yield and excellent enantioselectivity. Notably, it was confirmed that the halogen source and TsNH_2_ (the nitrogen source) co-generated the active species involved in the haloamination reaction, which is essential for the formation of key salt ion intermediates. A typical haloamine dependence was observed, demonstrating a sequential decrease in reactivity in the following order: NBS > NIS >> NCS. Additionally, by varying the substituents at the β-position of the substrate, similar experiments were conducted, yielding equally favorable outcomes (Scheme 32).

**Scheme 33:**
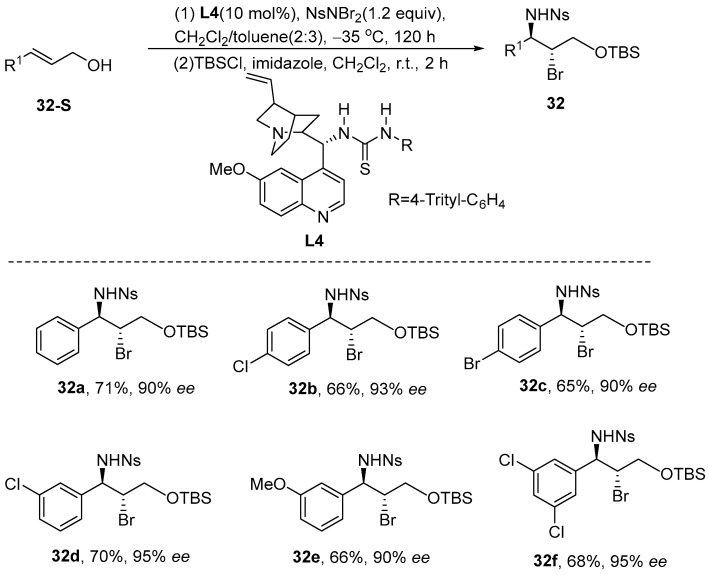
Enantioselective bromoamination reaction of allyl alcohol.

Qi et al. [83] first reported the enantioselective bromoamination of allyl alcohol in 2012. In their study, a cinchona-derived thiourea catalyst was employed in conjunction with N,N-dibromo-4-nitrobenzene sulfonamide, which served as both the bromine and amine source. The reaction was conducted in dichloromethane at 20 °C, resulting in products with moderate to good yields and excellent enantioselectivity (Scheme 33).

**Scheme 34:**
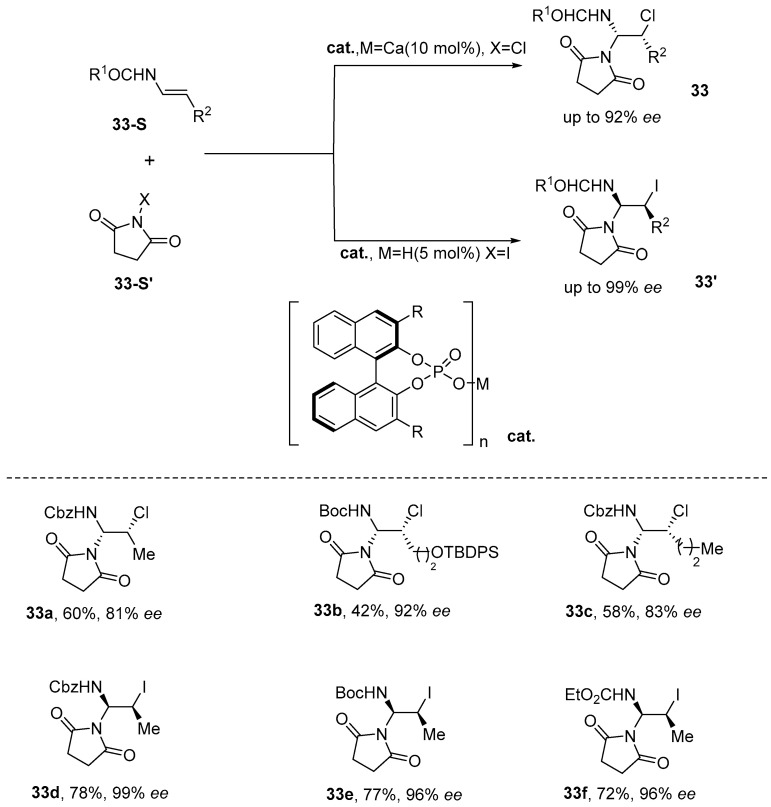
Chiral phosphoric acid or chiral calcium organophosphates catalyze intrahydanamide.

In 2016, Lebée et al. [84] introduced a catalytic methodology that enables the enantioselective vicinal iodoamination and chloroamination of enecarbamates. By employing chiral phosphoric acid or chiral calcium organophosphate as catalysts, a diverse array of enantiomerically enriched cis-iodilamides and cis-chloramines was synthesized, achieving excellent yields along with high diastereomeric and enantiomeric selectivity. Furthermore, the products underwent azidation, during which sodium azide directly replaced iodine, yielding good azide products without significant racemization (Scheme 34).

**Scheme 35:**
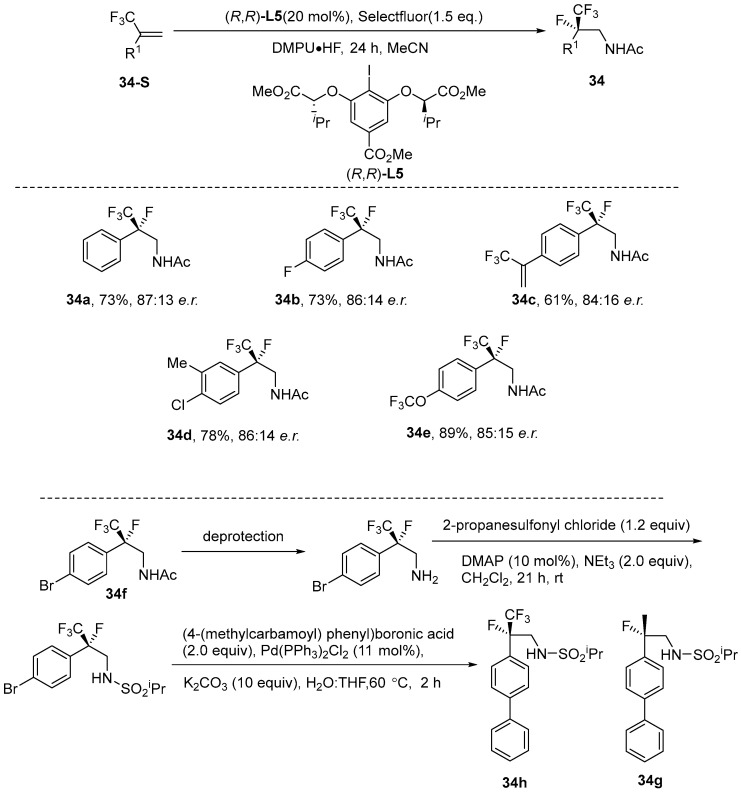
Iodine-catalyzed intermolecular enantioselective fluoroamination.

Schäfe et al. [85] proposed the utilization of C_2_-symmetric resorcinol-based aryl iodines(I/III) [86] as catalysts for the stereoselective intermolecular fluoroamination of α-trifluoromethyl styrenes. In this approach, a simple nitrile functions dually as both a solvent and a nucleophilic reagent, effectively intercepting iodine intermediates during the transition state. Consequently, this method produces amide products with yields of up to 89% (e.r. 93:7) and readily generates tertiary phenyl stereocenters containing CF_3_ and F groups. It is worth noting that product **34f**, after deprotection, can undergo a series of reactions to generate **34h**. **34h** is a CF_3_ analog of the potential Parkinson’s disease drug **34g** (Scheme 35).

### 4.3. Intermolecular Diastereoselective and Enantioselective Haloamination

**Scheme 36:**
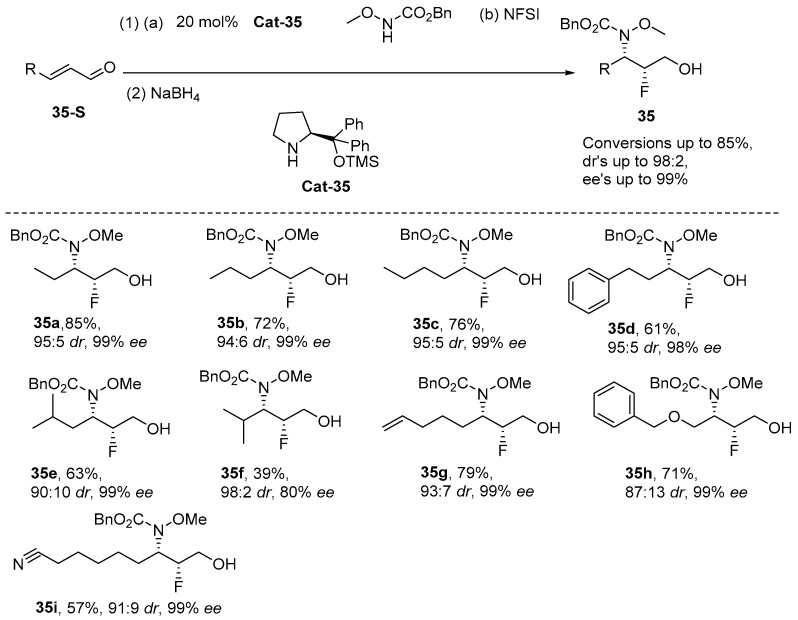
Organocatalytic enantioselective olefin fluoroamination.

Appayee et al. [87] reported the development of an organocatalytic stereoselective olefin fluoroamination reaction in 2010. Notably, this method facilitates the efficient synthesis of chiral α-fluoro-β-amino acids from simple achiral precursors, achieving excellent diastereomeric ratios (*drs*) and enantiomeric excesses (*ees*). This advancement establishes a robust foundation for further investigations into this organic transformation. The regioselectivity of α,β-unsaturated carbonyl compounds is primarily governed by the synergistic interplay between the substrate’s electronic properties and the catalyst’s steric effects. This system achieves precise control over reaction site selectivity through the formation of a rigid complex between the chiral organocatalyst and TsNHCl: the strongly electron-withdrawing carbonyl group renders the β-carbon significantly electron-deficient, promoting preferential nucleophilic attack by the nitrogen source at this position, while the chiral environment of the catalyst directs the fluorine source to selectively add to the α-position via steric hindrance, ultimately enabling efficient construction of α-fluoro-β-amine products. This characteristic “α-halo/β-amine” regioselectivity pattern stems from the catalyst’s multifaceted control over the reaction transition state—it simultaneously exploits the substrate’s inherent electronic effects and imposes spatial constraints through carefully designed chiral pockets, thereby achieving excellent regioselectivity and stereoselectivity (Scheme 36). The stereochemical pathway of the reaction is highly dependent on the synergistic interaction of the chiral ligand with TsNHCl to achieve antiselectivity by bridging the ammonium chloride ion intermediate. Fine tuning of the reaction conditions (such as temperature, additives) further optimized the stereocontrol (as shown in Table 1).

**Scheme 37:**
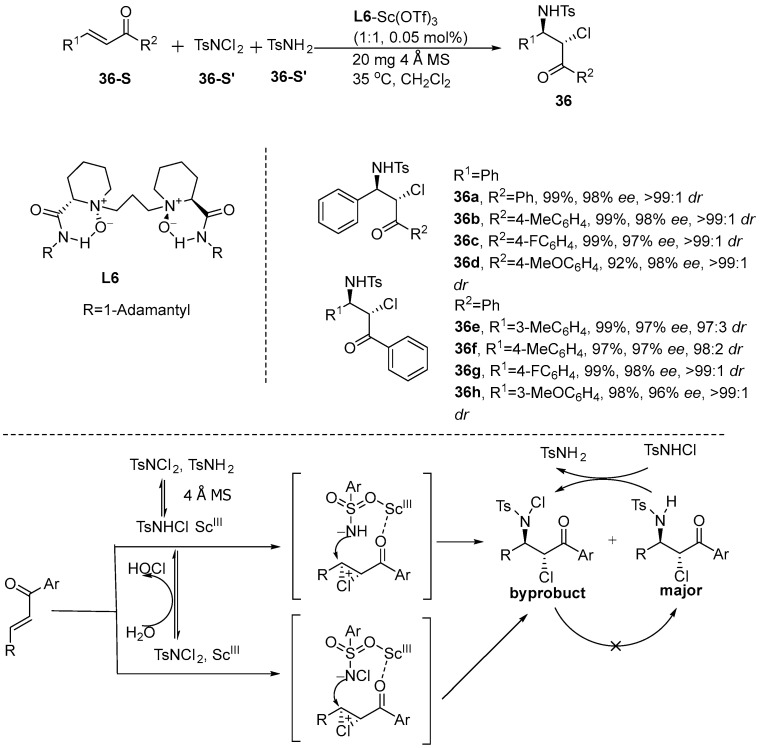
Stereoselective chloramination of chalcones.

In 2011, Cai et al. [88] developed a highly efficient stereoselective chloroamination reaction using α,β-unsaturated γ-ketone esters and chalcone as substrates and employing TsNH_2_/TsNCl_2_ (1:1) as sources of chlorine and nitrogen, achieving excellent yields along with superior diastereoselectivity and enantioselectivity. As shown in Table 2, the stereoselectivity of the reaction was synergistically controlled by the chiral catalyst and the reaction conditions. The reaction mechanism was as follows: under the promotion of 4 Å MS, highly active TsNHCl was generated, the formation of chiral ammonium chloride ion intermediates was promoted by the N,N’-dioxide-Sc(III) complex after negative nitrogen nucleophilic attack, and finally the product was generated, as well as trace byproducts (Scheme 37).

**Scheme 38:**
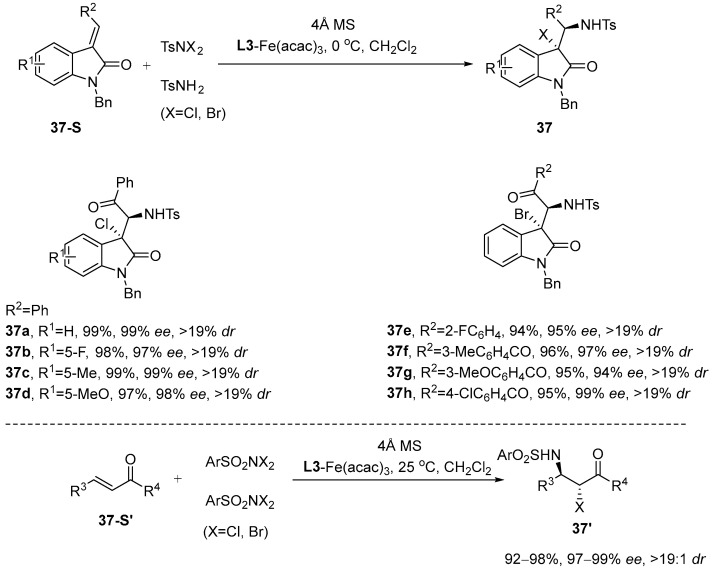
Intermolecular stereoselective haloamination catalyzed by iron transition metals.

Cai et al. [89] reported that chiral iron-based catalysts exhibited superior enantioselectivity in catalytic reactions compared to other transition metal catalysts, although this was observed only in select cases. Notably, Cai et al. successfully developed an intermolecular chloroamination and bromoamination of 3-enylindole using iron(III)/N,N’-dioxide complexes as catalysts. Their approach resulted in products with exceptionally high yields (99%) and remarkable enantioselectivity (99%) and diastereoselectivity (*dr* > 19:1) (Scheme 38). Table 3 presents the effects of reaction conditions on stereoselectivity.

**Scheme 39:**
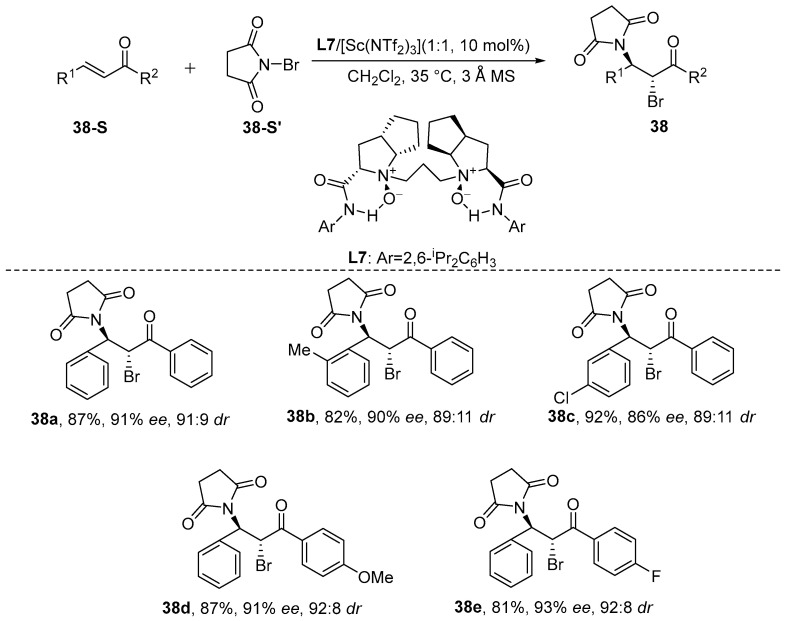
Chiral N,N’-dioxide-Sc(NTf_2_)_3_ complex catalyzes stereoselective bromoamination reaction of chalcone.

Wang et al. [90] reported, in 2017, a chiral N,N’-dioxide-Sc(NTf_2_)_3_ complex-catalyzed stereoselective bromoamination reaction of chalcone [91]. In their study, N-bromosuccinimide functioned as both the bromine and amide source. Under mild reaction conditions, the corresponding chiral brominated products were obtained in high yields (up to 92%) with an excellent diastereomeric ratio (*dr* value of 93:7) and outstanding enantiomeric excess (*ee* value of 97%) (Scheme 39). Table 4 explains the effect of reaction conditions on stereoselectivity.

## 5. Chiral Catalyst/Ligand Design: Influence on Stereoselectivity and Green Chemistry Performance

Table 5 provides a concise summary of the aforementioned reactions, elucidating the structural modifications of chiral catalysts/ligands and their impacts on stereoselectivity and green chemistry metrics, along with the specific green chemistry principles fulfilled by each reaction system.

## 6. Conclusions and Outlook

Herein, we systematically reviewed recent advances in stereoselective haloamination of olefins catalyzed by both chiral metal (Cu, Pd, Fe, etc.) and non-metal catalysts. While significant achievements have been made in this field, several critical challenges remain to be addressed: the difficulty in controlling enantioselectivity for fluoroamination reactions, limited substrate scope for complex molecules, harsh reaction conditions hindering industrial applications, and insufficient mechanistic understanding due to the lack of in situ characterization techniques. These limitations currently restrict the broader application of haloamination reactions in pharmaceutical synthesis and related fields.

To address these challenges, the integration of electrochemical approaches, continuous-flow technology, and AI-assisted catalyst design is expected to bring transformative breakthroughs. Electrochemical methods enable in situ generation and precise control of active halogen species, potentially replacing traditional oxidants (such as Lei et al. [92], who successfully achieved electrochemical iodoamination of various indole derivatives using a series of nonactivated amines, obtaining products with yields up to 98%). Continuous-flow technology offers enhanced mass/heat transfer in microreactors, improving process safety and scalability (such as Oliver et al. [93], who reported a metal-free, visible-light-mediated, continuous-flow protocol for the iodoamination of alkenes using commercially available NIS and protected amines). Meanwhile, AI-assisted catalyst design can accelerate reaction optimization and overcome selectivity limitations. The synergistic combination of these cutting-edge technologies may revolutionize conventional research paradigms in haloamination reactions, opening new avenues for pharmaceutical and functional materials synthesis. Although challenges remain in interdisciplinary integration, scale-up applications, and data standardization, these advanced strategies will undoubtedly facilitate the transition of stereoselective haloamination from fundamental research to industrial implementation.

## Figures and Tables

**Table 1:** Relationship between reaction conditions and stereochemical pathways.

Reaction Step	Key Condition	Stereochemical Control Mechanism	Selective Results
1. Imine-catalyzed conjugated addition	Catalyst: **Cat-35**	(1) The chiral center of 12a guides the nucleophilic reagent. (2) The silicon group provides steric hindrance shielding on the Si surface, enhancing the enantioselectivity.	*ee* content as high as 99%
	Solvent: MTBE (methyl tert-butyl ether)	Non-polar solvents protect **Cat-35** from being deactivated by NFSI through silicon removal, thereby maintaining its catalytic activity.	Improves *ee* and stability of intermediates during reaction process
	Temperature: rt	Room temperature facilitates the efficient completion of imine formation and amine addition.	Shortens reaction time and reduces side effects
2. Cycloammonium-catalyzed fluorination	Catalyst **Cat-35** (sustaining action)	(1) **Cat-35** reacts with saturated aldehyde to form enamine. The Si face is shielded by the silicon group, and fluorine attacks from the Re face. (2) There is an enhanced synselectivity at low temperature (0 °C).	*dr* 95:5 (syn/anti)
	Fluorine source: NFSI (N-Fluorobenzenesulfonimide)	The electrophilic fluorinating reagent selectively attacks the Re face of the alkenylamine, generating an α-fluorinated product.	High synselectivity
	Solvent: MTBE	A low-polarity environment reduces catalyst deactivation and avoids side reactions.	Maintains high *dr* and *ee*
	Temperature: 0 °C	(1) This enables the low-temperature inhibition of side reactions, fixation of enamine conformation, and enhancement of synselectivity. (2) A high temperature (rt) leads to a selective reversal (*dr* 3:1).	Optimizes synselectivity (95:5)

**Table 2:** Source analysis of enantioselectivity and diastereoselectivity.

Selective Type	Key Influence Factor	Embody	Mechanistic Explanation
Enantioselectivity (*ee*)	Chiral ligand: (S)-piperidine acid-derived N,N’-dioxygenated **L6**-Sc(OTf)_3_	*ee* up to 99%	Chiral ligands control the attack surface of TsNH_2_ by inducing the direction of formation of the chloroammonium ion intermediate through spatial site resistance and electronic effects.
	Reagent combination: TsNCl_2_/TsNH_2_ to generate highly reactive TsNHCl	Increase in *ee* from 94% to 97% with TsNCl_2_/TsNH_2_	TsNHCl synergizes with chiral catalysts to form chloroammonium ion intermediates in a single configuration to avoid racemization.
	Temperature: low (0 °C)	Heterocyclic substrates up to 99% *ee* at 0 °C	The low-temperature inhibition of intermediate racemization provides improved chiral control.
	Electronic effects: Substrate aromatic ring substituents	Higher *ee* for electron donor; slightly lower *ee* for electron absorber	The electron-donating group stabilizes the chloroammonium ion intermediate and reduces racemization; the electron-withdrawing group accelerates the dissociation of the intermediate and reduces selectivity.
Diastereoselectivity (*dr*)	Reaction mechanism: Bridging of ammonium chloride intermediate molecule	*dr* > 99:1	The bridging structure forces TsNH_2_ to attack from the opposite (anti-) side to form a single diastereomer.
	Substrate structure: Steric hindrance at ester group position	Yield of bulky ester group decreased but *dr* remained unchanged	Steric hindrance affects the spatial matching of the transition state, but the anti-addition pathway remains dominant.
	Electron effect: Aromatic ring substituent	Higher electron-donating group *dr*; slightly lower electron-absorbing group *dr*	The electron-donating groups enhance intermediate stability and reduce side pathways; electron-withdrawing groups may trigger competing reactions.
	Additive: 4 Å MS	With no 4 Å MS, *dr* remains unchanged but yield decreases	4 Å MS inhibits the side reactions by removing water, but does not affect the stereochemistry of the main pathway.

**Table 3:** The relationship between reaction conditions and stereoselectivity.

Influence Factor	Enantioselectivity (*ee*)	Diastereoselectivity (*dr*)
Chiral catalyst (**L3**–Fe(acac)_3_)	Provides chiral environment to direct absolute configuration of product	The stereochemistry of the transition state is controlled by the ligand, achieving high diastereoselectivity (*dr* > 19:1).
Halogenating reagent (TsNCl_2_/TsNH_2_)	Enantioselective attack of reactive intermediates	The steric and electronic effects of the reagents influence the ratio of diastereomers.
Reaction temperature (0 °C or 25 °C)	Low temperature reduces side effects and improves *ee* value	The low temperature suppresses the competitive formation of diastereomers, thereby enhancing the diastereomeric ratio (*dr*).
Solvent (CH_2_Cl_2_)	Stabilizes intermediates by providing suitable polar environment	The polarity and coordinating ability of the solvent modulate the energy difference between transition states.
4 Å MS	Avoids water induced catalyst deactivation and maintains high *ee* values	The 4 Å MS reduces side reactions and maintains high *dr*.
Substrate structure	Electronic effects and steric hindrance influence efficiency of chiral induction	The steric hindrance of the substituents determines the ratio of the diastereomers.

**Table 4:** Reaction conditions with respect to diastereoselectivity (*dr*) and enantioselectivity (*ee*).

Reaction Condition	Enantioselectivity (*ee*, %)	Diastereoselectivity (*dr*, ratio)	Possible Influencing Factors
Standard conditions (NBS, Sc(NTf_2_)_3_)	90–94% (most > 90%)	88:12 to 92:8 (most > 90:10)	The efficient control of stereochemistry by a chiral catalyst (**L7**-Sc(NTf_2_)_3_); the dual role of NBS as a bromine and amide source.
NIS replaces NBS	73%	96:4	The larger size of the iodine atom may reduce the spatial matching of the transition state, but it has less effect on the diastereoselectivity.
NCS replaces NBS	69%	85:15	The high electronegativity but small size of the chlorine atom may lead to a weakened chiral induction and a slight decrease in diastereoselectivity.
Base-free conditions	Trace	-	Base absence deactivates the intermediate, preventing reaction.
With TEA (as base)	75%	-	TEA facilitates the formation of intermediates, but excessive alkalinity may interfere with chiral control.
With DIPA (as base)	33%	-	The strong base (DIPA) disrupts the chiral environment, leading to a significant reduction in enantioselectivity.

**Table 5:** Structural modifications of chiral catalysts/ligands and their impact on stereoselectivity and green chemistry.

Catalyst/Ligand	Representative Structure/Modification	Reaction System	*ee* (%)	*dr*	Green Chemistry Metrics	Green Chemistry Principles
Photocatalysis [39]	Organic photosensitizer	NIS, visible light, green solvent	-	-	Metal-free, visible-light-driven, biodegradable solvent, room-temperature reaction	**HH**, **DD**, **EE**
Aminothiocarbamate [41]	Thiourea scaffold, Quinoline moiety	NBS, CH_2_Cl_2_, −78 °C	99%	-	High atom economy, metal-free	**II**, **EE**
C_2_-Symmetric Selenium-Based Catalyst [43]	Selenium-embedded cyclic structure	NBP, solvent-free	>90%	-	Metal-free and highly active	**FF**
Cu(II)–bisoxazoline complexes [44]	Sterically demanding aryl groups (phenyl, tert-Butyl)	Cu(II)-catalyzed manganese dioxide oxidation, CH_2_Cl_2_	>90%	-	Broad substrate scope	**GG**
BINAP(S) [46]	Mono-thioBINAP derivative	NBS, toluene/CH_2_Cl_2_, −78 °C, 50 h	>90%	-	High stereoselectivity	**CC**, **II**
C_2_-Symmetric Bisamidine (BAM) [49]	Nitrogen electron modulation, sterically bulky substitution	NIS, metal-free, mild Conditions	>90%	-	Metal-free, high N-selectivity, derivatizable	**AA**, **BB**, **CC**, **DD**
Metal-free [50]	Chiral iodoarenes	Fluoroamination, chiral iodoarenes	>90%	-	Metal-free	**EE**
(S,S)-Cat-Mn [52]	Chiral N-pyridinium ion intermediates	NaClO/NaBrO, PhCl, pH 11–11.5, 10 °C	99%	-	Neutral pH, mild temperature	**DD**, **FF**
K_3_PO_4_ Catalysis [76]	Maintaining rigid transition state of reaction intermediates	BocNBr_2_/BocNH_2_, metal-free	-	23:1	Metal-free, mild deprotection	**DD**, **EE**
**L3**-Sc(OTf)_3_ [81]	Diisopropylamino-modified Sc(III) complex	NIS/TsNH_2_, 4 Å MS, light-free	>90%	-	Bio-based catalyst	**GG**, **FF**
Cinchona alkaloid-thiourea [82]	Natural base-derived hydrogen bond donor	N,N-Dibromosulfonamide, CH_2_Cl_2_, 20 °C	>90%	-	Low catalyst loading	**FF**, **DD**

**AA—**the principles of low-toxicity synthesis; **BB—**byproduct minimization; **CC—**the design of safer chemicals; **DD—**energy efficiency optimization; **EE—**the principle of selecting safer chemical substances; **FF—**high-performance catalyst application; **GG—**the principles of renewable feedstock utilization; **HH—**the principles of safer solvents; **II—**the principles of atom economy maximization.

## Data Availability

No new data were created.
